# YNbC, novel Janus MXene: a first-principles study

**DOI:** 10.1039/d6ra06232f

**Published:** 2026-10-20

**Authors:** Hamze Mousavi, Moein Asshabi

**Affiliations:** a Department of Physics, Razi University Kermanshah Iran hamze.mousavi@gmail.com

## Abstract

The unique out-of-plane structural asymmetry of Janus MXenes makes them an attractive class of two-dimensional materials, enabling flexibility in tailoring their physical properties. Employing first-principles calculations, we systematically explored the structural stability, mechanical properties, electronic structure, and thermoelectric transport properties of the novel Janus YNbC monolayer in comparison with its parent Nb_2_C and Y_2_C MXenes. The mechanical and dynamic stability of the proposed structure was established through elastic-constant analysis and phonon-dispersion spectra, respectively. Cohesive-, formation-, and decomposition-energy analyses indicate favorable bonding with respect to isolated atoms and a metastable character relative to the considered parent-MXene phases. Its finite-temperature thermodynamic behaviour was investigated within the harmonic phonon approximation. The calculated mechanical properties indicate that YNbC possesses favorable mechanical robustness with characteristics intermediate between those of Nb_2_C and Y_2_C. Electronic-structure calculations reveal a substantial modification induced by Janus symmetry breaking. While the parent MXenes exhibit metallic characteristics, at the PBE level, Janus YNbC shows semimetallic behaviour, characterized by a small indirect band overlap of approximately 14.6 meV, whereas the HSE functional produces a finite indirect band gap of approximately 0.1 eV, indicating narrow-gap semiconducting behaviour at this level of theory. Furthermore, the calculated thermoelectric transport coefficients demonstrate a favorable thermoelectric response for YNbC compared with the parent MXenes. Overall, Janus engineering offers a promising route to transforming metallic MXenes into narrow-gap materials with favorable electronic thermoelectric transport properties. These results suggest that narrow-bandgap YNbC holds promise for electronic and thermoelectric applications.

## Abstract 2

The unique out-of-plane structural asymmetry of Janus MXenes makes them an attractive class of two-dimensional materials, enabling flexibility in tailoring their physical properties.

## Graphical abstract

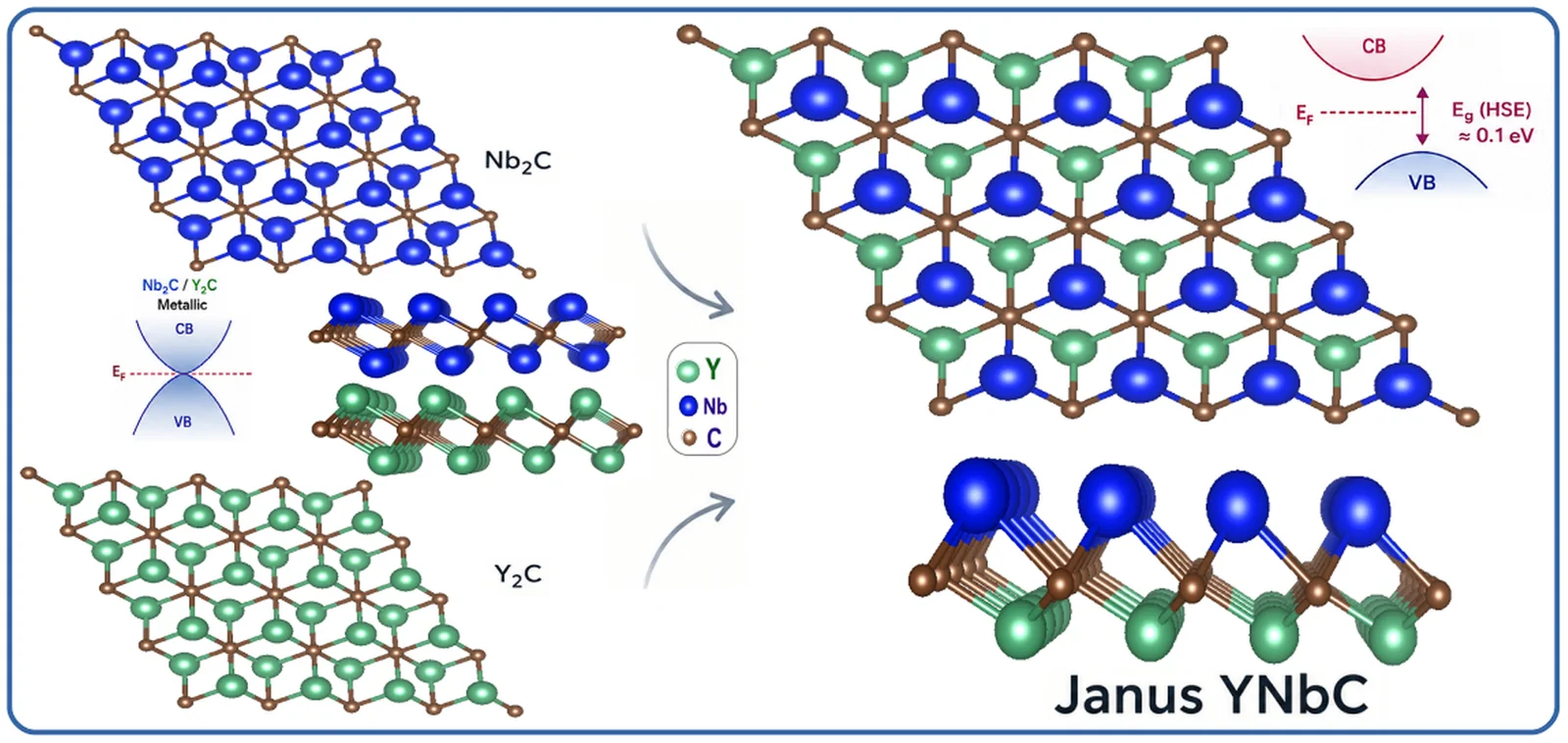

## 1 Introduction

Since their first experimental realization by Barsoum and co-workers,^1–3^ MXenes have become one of the most extensively investigated families of two-dimensional (2D) materials. They have attracted tremendous attention alongside graphene, hexagonal boron nitride, and transition-metal dichalcogenides such as MoS_2_.^4–7^ Given the promising prospects of 2D systems, significant efforts are underway in their synthesis and computational prediction,^8,9^ and researchers are actively investigating the properties of this class of materials.^10,11^ MXenes, in particular, have received significant research attention owing to their promising properties and potential as a class of atomically thin materials.

MXenes are obtained by removing the element A from the 3D chemical compositions of MAX phases with the space group *P*6_3_/*mmc* and general chemical formula M_*n*+1_AX_*n*_. After removing element A from the above-mentioned compound, monolayers of MXene with chemical formula M_*n*+1_X_*n*_ and space group *P*3̄*m*1 are obtained. In the previously discussed chemical compounds, M denotes a transition-metal element, A denotes an element from groups 13–16, X represents either carbon, nitrogen, or both elements, and *n* takes the integer values 1, 2, or 3.^1–3^ Following their introduction and characterization, MXenes have been extensively studied for their diverse properties and applications. Among the various applications of MXenes, the following examples are noteworthy. MXenes have recently emerged as highly attractive materials for the electrocatalytic conversion of carbon dioxide (CO_2_), owing to their chemically tunable surfaces, high specific surface area, and high electrical conductivity. While pristine MXenes exhibit limited catalytic performance, their composites and modified forms have shown enhanced activity, particularly in electrocatalytic CO_2_ reduction reactions. Moreover, the development of MXene quantum dots has introduced new possibilities for CO_2_ conversion, especially through photocatalytic and chemical pathways.^12,13^ The outstanding electrochemical behaviour of MXenes has established them as attractive electrode materials for energy storage devices, particularly supercapacitors. Recent experimental investigations have explored synthesized MXenes as electrode materials through detailed electrochemical characterization. The pristine MXene electrodes exhibited high specific capacitance together with outstanding cycling durability. After 10 000 charge–discharge cycles, nearly 85% of their initial capacitance was retained. Overall, these results highlight the promise of MXenes for next-generation energy storage technologies.^14^ Moreover, in a computational study by Tatan *et al.*, the superconducting properties of functionalized MXenes Zr_2_NS_2_, Zr_2_NCl_2_, and Sc_2_NCl were examined using density functional theory (DFT) calculations.^15^ These practical applications underscore the importance of theoretical and computational studies to better understand the structure–property relationships and to design materials with enhanced performance. Following the extensive investigation of the diverse properties and applications of MXenes, researchers have sought to enhance their characteristics and achieve improved functionalities. One promising approach for tuning the properties of MXenes involves the design of Janus structures derived from these 2D layers.

In 2D Janus materials, the atomic arrangement is purposefully modified so that the top and bottom surfaces of a single monolayer consist of different chemical species or environments. This structural asymmetry breaks the vertical mirror symmetry characteristic of conventional 2D crystals.^16,17^ The broken structural symmetry gives rise to an intrinsic dipole moment together with a built-in electric field across the monolayer. Relative to their symmetric counterparts, including MoS_2_ and Ti_2_C, Janus monolayers exhibit distinctive structural and electronic characteristics, including modified bond lengths, redistributed charge density, and out-of-plane polarity. These differences directly influence their physical properties and make Janus 2D layers well suited for applications in nanoelectronics and optoelectronics.^16^

The advent of 2D Janus materials, particularly TMDs, has prompted extensive research into their unique electronic, optical, and structural properties arising from out-of-plane asymmetry. Recent studies using first-principles calculations on van der Waals heterostructures built on Janus MXY monolayers (M = Mo, W; X, Y = S, Se, Te; X ≠ Y) have revealed power conversion efficiencies exceeding 20%, along with tunable electronic structures influenced by spin–orbit coupling and intrinsic dipole effects.^18^ In addition, the fabrication of nanoscrolls from Janus MoSSe and WSSe, as well as plasma-assisted growth techniques, has demonstrated enhanced optical responses and photoluminescence, while simulated Raman spectra provide predictive fingerprints for experimental identification and structural characterization.^19–21^

Given the above, the prediction of Janus structures in 2D materials has attracted considerable attention, prompting extensive theoretical and experimental investigations. In this context, MXenes offer an attractive platform for the theoretical prediction and synthesis of Janus MXene structures. Recent theoretical and computational studies on Janus MXenes have revealed diverse characteristics and promising applications in energy storage, magnetism, and photocatalysis. First-principles calculations indicate that specially designed Janus MXene monolayers incorporating different transition metals on opposite surfaces serve as efficient host materials for Na metal anodes, where the intrinsic structural asymmetry enhances sodiophilicity and cycling stability relative to symmetric MXenes.^22^ Moreover, DFT studies on double transition metal Janus MXenes of the type MCrCT_2_ (M = Ti, Mo; T = O, F, OH) have demonstrated a variety of electronic behaviours, ranging from metallic and semimetallic characteristics to fully half-metallic states with high spin polarization, as well as intrinsic ferromagnetism that is attractive for future spintronic and nanoelectronic applications.^23^ Additional computational work has explored the structure, magnetism, and electronic behaviour of oxygen-functionalized Janus MXene CrScCO_2_, revealing robust ferromagnetic ordering with half-metallic behaviour in most configurations, indicating potential utility in spintronic devices.^24^ DFT studies have also examined multivalent ion storage in Janus Zr_2_CSSe MXene, showing that the built-in electric field due to asymmetry reduces diffusion barriers and leads to high theoretical capacities for Na, Mg, and Ca ions, pointing to broad applicability in next-generation battery technologies.^25^ In parallel, novel Janus MXene YZrNOS has been investigated for photocatalytic water splitting, where high visible-light absorption capability and favorable carrier mobility suggest exceptional potential for solar-to-hydrogen conversion applications.^26^ Beyond energy and charge transport, strain-induced phenomena have been reported in Janus MXene nanosheets such as TiVC, where theoretical calculations indicate a reversible magnetic state transition from antiferromagnetic to ferromagnetic ordering under strain, enabling solid-state information storage without external power.^27^ These diverse computational efforts demonstrate that compositional tuning, surface functionalization, and strain engineering of Janus MXene systems provide a valuable framework for tuning electronic, magnetic, and energy-related properties in 2D monolayers.

Despite the extensive research conducted on Janus MXenes, the proposed Janus YNbC monolayer (j-YNbC) has not yet been investigated. According to the available literature, its stability and physical characteristics have not yet been comprehensively investigated. In particular, its energetic, dynamical, mechanical, and ground-state stability, as well as its phononic, thermodynamic, electronic, and thermoelectric characteristics, remain to be systematically investigated. Furthermore, the influence of Janus engineering on these properties has not been systematically compared with those of the corresponding parent MXenes. Therefore, a comprehensive first-principles investigation is required to assess its structural, physical, and thermoelectric properties and to evaluate its potential for future applications. Motivated by the growing interest in Janus 2D materials and the distinctive characteristics arising from structural asymmetry, we perform first-principles DFT calculations to examine the proposed j-YNbC monolayer together with its parent MXenes, Y_2_C and Nb_2_C. First, their structural characteristics and various stability criteria, including energetic, dynamical, mechanical, and ground-state stability, are systematically examined. Subsequently, the phononic and thermodynamic properties are systematically investigated. The mechanical behaviour, electronic structure, and thermoelectric performance are subsequently examined in detail. A comparative analysis of j-YNbC and its parent MXenes is performed to clarify how Janus engineering influences their physical characteristics. This comprehensive study provides deeper insight into the structure–property relationships of the proposed MXene systems and evaluates their potential for future technological applications.

## 2 Computational details

First-principles DFT calculations were carried out using the Quantum ESPRESSO (QE) package.^28–30^ The generalized gradient approximation (GGA) with the Perdew–Burke–Ernzerhof (PBE) functional was employed to describe the exchange–correlation effects.^31^ For j-YNbC, additional single-point calculations were carried out using the HSE06 hybrid functional to obtain a more reliable description of its near-Fermi-level electronic structure and band gap.^32,33^ The same pseudopotentials were used for both the PBE and the HSE calculations. The standard solid-state pseudopotential (SSSP) library version 1.3.0 was employed for the electron–ion interaction.^34,35^ The valence electron configurations considered in this work are as follows: Y: 4s^2^4p^6^4d^1^5s^2^5p^0^, Nb: 4s^2^4p^6^4d^4^5s^1^5p^0^, and C: 2s^2^2p^2^. For the spin–orbit coupling (SOC) calculations, fully relativistic pseudopotentials from the PSLibrary 1.0.0 were employed.^36^ The kinetic energy cutoff for the plane-wave basis set was set to 55 Ry, whereas the charge-density cutoff was fixed at 440 Ry (corresponding to eight times the wave-function cutoff). To prevent interactions between periodically repeated 2D layers, a vacuum spacing of at least 15 Å was applied along the *z*-direction. A *Γ*-centered 20 × 20 × 1 Monkhorst–Pack *k*-point mesh^37^ was employed for the majority of the PBE calculations. Convergence tests indicated that coarser *k*-point meshes also provided satisfactory accuracy; accordingly, a 16 × 16 × 1 *k*-point mesh was adopted for the phonon calculations to provide an appropriate balance between computational cost and accuracy. The dynamical matrices were computed on an 8 × 8 × 1 *q*-point grid within density functional perturbation theory (DFPT). For the HSE calculations, a denser 24 × 24 × 1 *k*-point mesh was adopted to improve the resolution of the near-Fermi-level electronic structure while accounting for the computational limitations of the hybrid-functional calculations. In addition, a 4 × 4 × 1 *q*-point mesh was used for the Fock-exchange calculations within HSE, commensurate with the *k*-mesh (ratio 6 : 1). This coarser *q*-grid was adopted because the computational cost of the exact-exchange evaluation scales steeply with the *q*-point density, and a *q*-mesh coarser than the one used for the self-consistent density and potential is generally regarded as adequate for Fock-exchange evaluation in the literature.^30^ Given the computational cost associated with the 24 × 24 × 1 *k*-mesh combined with the hybrid-functional Fock-exchange evaluation, the 4 × 4 × 1 *q*-mesh represented the highest density practically accessible with the available computational resources. The resulting phonon spectra were subsequently employed to evaluate the finite-temperature vibrational thermodynamic properties within the harmonic phonon approximation using the QHA code distributed with QE.^30,38^ A single force-constant matrix obtained at the optimized equilibrium structure was used throughout the calculations, without considering temperature-dependent lattice expansion. The structures were fully optimized until the total energy converged to within 1.0 × 10^−5^ Ry and the atomic forces fell below 1.0 × 10^−4^ Ry Bohr^−1^. The electronic self-consistent field calculations were converged to a total-energy threshold of 1.0 × 10^−8^ Ry. The required supercells for the magnetic calculations were generated using the Phonopy code.^39^ Subsequently, biaxial strains ranging from ±7% with increments of 1% were applied, and the atomic positions were fully relaxed at each strain level. Details of the magnetic configurations are presented in the Results section. The elastic constants and derived mechanical properties were obtained using a custom Python code developed by the authors. The BoltzTraP2 code was employed to evaluate the thermoelectric properties within the constant-relaxation-time approximation (CRTA).^40^ No specific numerical value of the carrier relaxation time *τ* was assigned; therefore, the electrical conductivity, electronic thermal conductivity, and power factor are reported in the normalized forms *σ*/*τ*_0_, *κ*_e_/*τ*_0_, and PF/*τ*_0_, respectively. The same CRTA framework was consistently applied to j-YNbC, Y_2_C, and Nb_2_C, allowing their normalized electronic transport properties to be directly compared. For the PBE-based electronic-structure and transport calculations, non-self-consistent calculations were performed on a 100 × 100 × 1 *k*-point mesh, and the resulting electronic bands were used as input for BoltzTraP2. An interpolation factor of 20 was employed to obtain a sufficiently dense representation of the electronic structure for the transport calculations. For the HSE-based transport calculations of j-YNbC, the HSE electronic bands obtained using the 24 × 24 × 1 *k*-point mesh were directly used as input for BoltzTraP2, with an interpolation factor of 100. No scissor correction was applied to the HSE bands. The transport coefficients were evaluated at temperatures of 300, 600, and 900 K.

## 3 Results and discussion

The results of the first-principles calculations are presented and discussed. The stability and structural characteristics of the investigated monolayers are examined first, followed by analyses of their phononic, thermodynamic, mechanical, electronic, and thermoelectric properties. Comparative discussions are provided throughout to elucidate the influence of Janus engineering relative to the corresponding parent MXenes.

### 3.1 Stability and structural properties

The optimized structures of the studied MXene and Janus monolayers are shown in Fig. 1. Fig. 1(a) shows the optimized crystal structures of the parent MXenes (M_2_C, M = Y and Nb) from the top and side perspectives. Both materials belong to the *P*3̄*m*1 space group (no. 164). Fig. 1(b) presents the j-YNbC monolayer, which crystallizes in the *P*3*m*1 space group (no. 156). Both structures are composed of three atomic layers, with a carbon layer located between two transition-metal layers. In contrast to the symmetric parent MXenes, the Janus structure contains different transition-metal atoms on its two outer surfaces. The asymmetric arrangement of the outer transition-metal atoms removes the inversion symmetry, resulting in the characteristic Janus configuration.

**Fig. 1:**
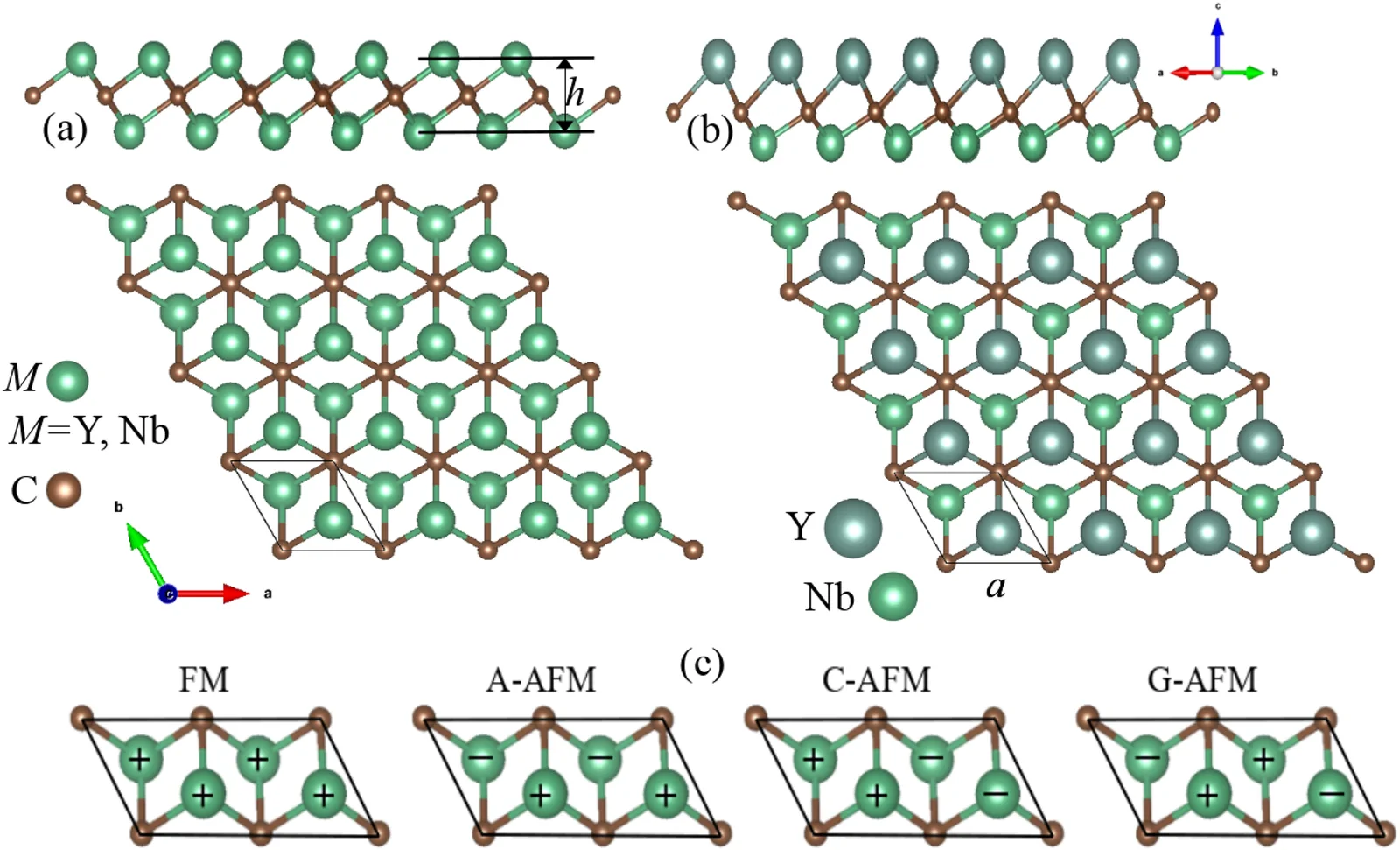
Side and top views of the optimized crystal structures of (a) M_2_C (M = Y, Nb) MXenes, and (b) the j-YNbC. (c) Schematic representation of the magnetic configurations considered in this work, including FM, A-AFM, C-AFM, and G-AFM states. The symbols “+” and “−” denote spin-up and spin-down magnetic moments, respectively.

Fig. 1(c) presents the 2 × 1 × 1 supercell employed for the magnetic ground-state calculations. Considering the number and chemical species of atoms in the primitive cell, only a single antiferromagnetic (AFM) arrangement can be defined. To explore additional magnetic orderings, a 2 × 1 × 1 supercell was constructed, enabling the investigation of three distinct A-, C-, and G-AFM antiferromagnetic configurations,^41^ together with the ferromagnetic (FM) and nonmagnetic (NM) states. The corresponding magnetic configurations are illustrated in Fig. 1(c). For each strain condition, complete structural optimization was performed prior to evaluating the total energies of the investigated magnetic configurations. The resulting energy-strain curves for j-YNbC are presented in Fig. 2. All curves exhibit a clear minimum at zero strain, indicating that the optimized structure corresponds to an equilibrium configuration and providing evidence of its structural stability against small in-plane strains.

**Fig. 2:**
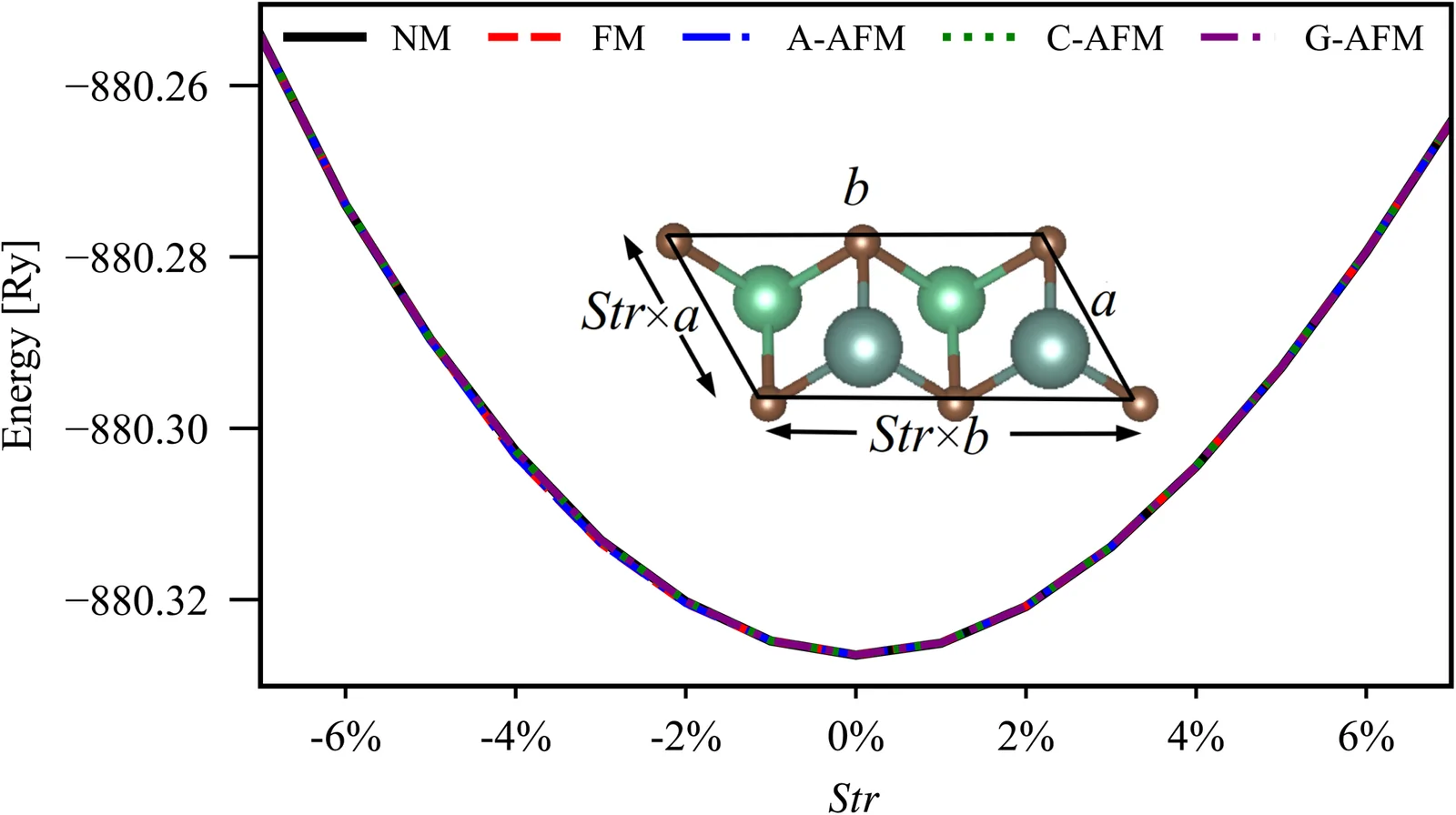
Total energy of the j-YNbC monolayer as a function of biaxial strain for different magnetic configurations. The inset shows the (2 × 1 × 1) supercell used in the calculations and schematically illustrates the applied biaxial strain.

For both j-YNbC and Nb_2_C, the NM configuration has the lowest total energy among all the considered magnetic states at the equilibrium point (zero strain). The magnetic configurations are nearly degenerate with the NM state, with energy differences of less than 0.13 meV per atom. Moreover, no net magnetic moment is obtained for the investigated magnetic configurations at the equilibrium structure. Thus, within the accuracy of the present calculations, the NM state is marginally favored energetically for both systems and was adopted as the ground-state configuration for the subsequent analyses. For Y_2_C, spin-polarized calculations reveal that the G-type antiferromagnetic (G-AFM) state has the lowest total energy in the 2 × 1 × 1 supercell, lying approximately 57 meV (9.5 meV per atom) below the NM state. However, the associated local magnetic moment is extremely small (0.05*µ*_B_ per Y atom, as obtained from Löwdin population analysis), indicating negligible magnetic polarization. Considering this very small magnetic moment together with previous spin-polarized theoretical studies on 2D Y_2_C reporting an effectively nonmagnetic ground state, as well as earlier first-principles studies that treated Y_2_C within a nonmagnetic framework,^42–46^ the NM configuration was therefore adopted for all subsequent analyses. The structural characteristics of the investigated compounds in their ground states are presented in Table 1 and discussed in detail in the following sections.

**Table 1:** Structural parameters, including the lattice constant (*a*), monolayer thickness (*h*), bond lengths (*d*_M–C_ and *d*_M′–C_) [Å], and cohesive energy (*E*_coh_) [eV per atom] for Y_2_C, Nb_2_C, and the j-YNbC monolayer

Structure	*a*	*h*	*d*_M–C_	*d*_M′–C_	*E*_coh_	Ref.
Y_2_C	3.5481	2.703	2.4544	—	−6.08	This work
	3.55	2.70	—	—	—	43
	3.51	—	2.44	—	−6.08	42
Nb_2_C	3.1197	2.37	2.15	—	−9.51	This work
	3.11	2.37	2.15	—	—	64
	3.1119	2.49	2.154	—	—	52
j-YNbC	3.293	2.567	(Nb–C)2.133	(Y–C)2.485	−7.77	This work

As illustrated in Fig. 3, no negative phonon branches or imaginary frequencies are observed throughout the Brillouin zone, confirming the dynamical stability of the investigated structures. The calculated elastic constants, listed in Table 2, confirm the mechanical stability of the 2D layers. According to the Born–Huang criteria for 2D hexagonal structures, stability requires *C*_11_ > |*C*_12_| and *C*_66_ > 0.^47^ Our results satisfy these conditions, confirming that all three layers are mechanically stable. The cohesive energies (*E*_coh_) were calculated using eqn (1), and the calculated values are listed in Table 1. The negative values of *E*_coh_ for all three monolayers indicate that their formation from isolated constituent atoms is energetically favorable.1
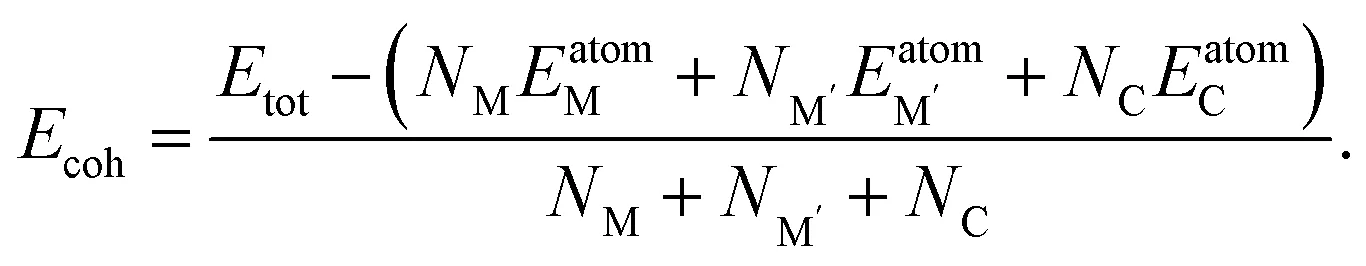


**Fig. 3:**
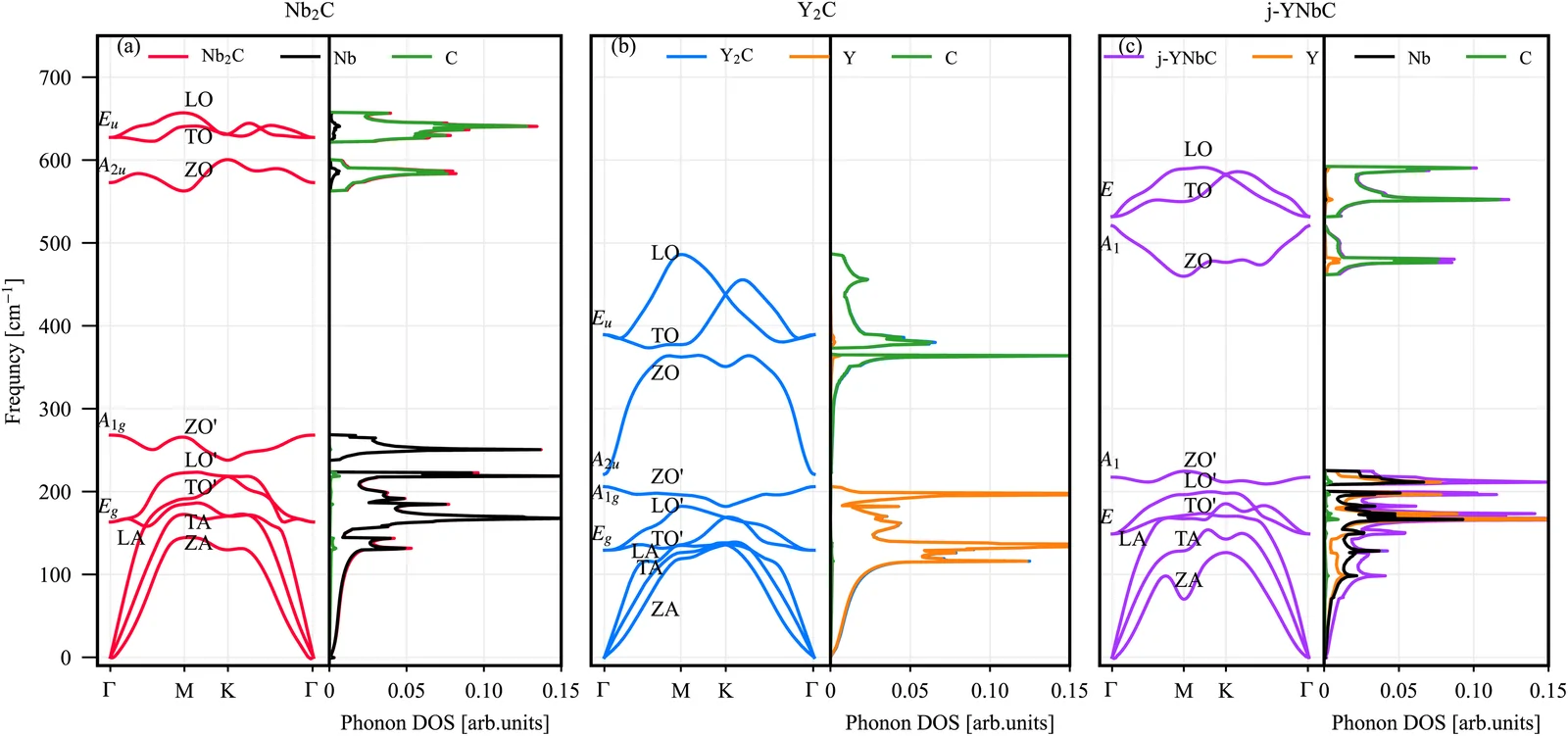
Phonon dispersion and phonon density of states (PhDOS), including total and projected contributions, for the Nb_2_C, Y_2_C, and j-YNbC monolayers shown in (a, b and c), respectively.

**Table 2:** Elastic constants (*C*_*ij*_), Young's modulus (*Y*_2D_), shear modulus (*G*), and two-dimensional bulk modulus (*B*_2D_) [N m^−1^], Poisson's ratio (*ν*) [dimensionless], and the bulk-to-shear modulus ratio (*B*_2D_/*G*) [dimensionless] of graphene, SL-BN, SL-MoS_2_, Y_2_C, Nb_2_C, and the j-YNbC monolayer

Structure	*C*_11_ = *C*_22_	*C*_12_	*C*_66_ = *G*	*ν*	Y_2D_	*B*_2D_	*B*_2D_/*G*	Ref.
Graphene	355.35	63.65	145.85	0.179	343.95	209.50	1.436	This work
	358.9	65.10	146.9	0.18	347.1	212.0	—	57
	366.9	66.6	145.95	0.20	361.75	216.72	1.46	65
SL − BN	290.72	64.65	113.03	0.22	276.34	177.69	1.572	This work
	289.8	63.7	113.1	0.22	275.8	176.8	—	56
SL − MoS_2_	132.65	32.56	50.04	0.245	124.65	82.60	1.650	This work
	132.3	32.8	49.5	0.25	124.1	82.50	—	57
Y_2_C	85.05	18.05	33.5	0.21	81.22	51.55	1.539	This work
	85.45	17.82	33.81	0.20	81.73	51.64	—	44
Nb_2_C	172.54	68.69	51.92	0.398	145.19	120.61	2.322	This work
	172.78	66.84	—	0.39	146.85	119.70	—	52
j-YNbC	134.79	39.768	47.51	0.295	123.06	87.28	1.837	This work

However, cohesive energy with respect to isolated atoms alone does not establish thermodynamic stability against decomposition into competing phases. Therefore, to distinguish the energetic favorability of bonding relative to isolated atoms from the stability with respect to condensed elemental phases, we additionally evaluated the formation energies of the investigated systems using the corresponding elemental reference states. In this analysis, *E*^bulk^_Y_, *E*^bulk^_Nb_, and *E*^graphite^_C_ denote the energies per atom of bulk hcp-Y, bulk bcc-Nb, and graphite, respectively, which were taken as the elemental reference states. Here, *N*_Y_, *N*_Nb_, and *N*_C_ represent the corresponding numbers of Y, Nb, and C atoms, while *E*_tot_ denotes the total energy of the considered monolayer. The formation energy per atom was calculated as2
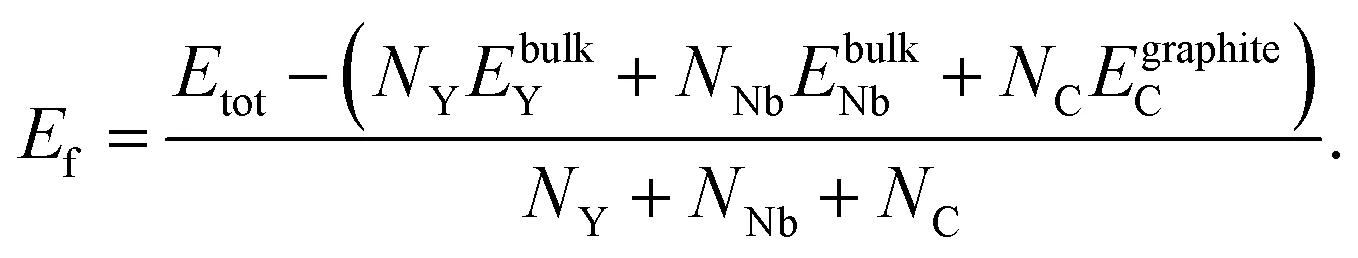


For graphite, the PBE + Grimme-D3 correction was employed to account for the interlayer interactions. The calculated formation energies of j-YNbC, Y_2_C, and Nb_2_C are +0.237, +0.076, and +0.351 eV per atom, respectively. The *E*_f_ of j-YNbC falls within the energy range defined by its parent MXenes, with a higher value than that of Y_2_C but a lower value than that of Nb_2_C. This places j-YNbC at an intermediate energetic level relative to the corresponding elemental reference states. However, these formation energies are defined with respect to the elemental phases and therefore cannot, by themselves, establish thermodynamic stability against competing compounds. To further assess the energetic stability of j-YNbC against relevant competing phases, its energy was compared with those of the corresponding parent MXenes, Y_2_C and Nb_2_C. In particular, the decomposition pathway
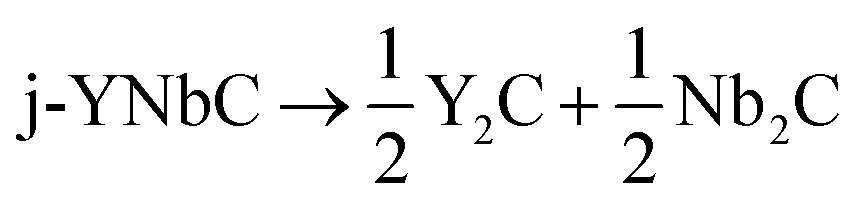
was considered. The corresponding decomposition energy was calculated as3
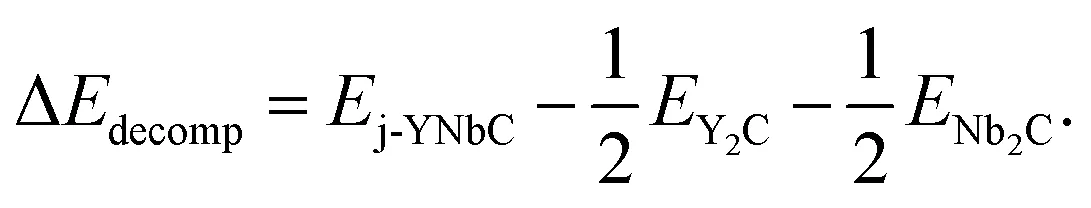


The calculated decomposition energy is approximately +23.6 meV per atom, indicating that j-YNbC lies only slightly higher in energy than the corresponding combination of its parent MXenes. Thus, j-YNbC can be regarded as metastable with respect to this specific decomposition pathway. It is worth noting that metastability is not uncommon among MXene systems.^48,49^ A systematic study by Ashton *et al.* showed that many MXenes lie above their corresponding competing bulk phases in energy and are therefore best regarded as metastable rather than thermodynamically stable phases.^50^ Importantly, several experimentally synthesized MXenes, including Nb_2_C, Ti_2_C, V_2_C, Ti_3_C_2_, Nb_4_C_3_, and Ta_4_C_3_, were identified within the metastable energy range.^51^ This demonstrates that a positive energy relative to competing phases does not necessarily preclude the experimental realization of an MXene. In the present case, considering the dynamical and mechanical stability, the presence of a well-defined energy minimum, and the relatively small decomposition energy with respect to the considered pathway, j-YNbC can be regarded as a metastable phase.

In addition to the *E*_coh_ discussed above, Table 1 summarizes the structural parameters of the investigated 2D layers, namely lattice parameters (*a*), bond lengths (*d*_C–M,M′_), and thickness (*h*). Our calculated values for pristine Nb_2_C and Y_2_C agree well with the available literature, supporting the validity of the present calculations. The *a* and *h* of j-YNbC (3.293 Å and 2.567 Å, respectively) lie between those of Y_2_C (3.54 Å and 2.7 Å) and Nb_2_C (3.12 Å and 2.37 Å). Notably, in j-YNbC, the bond length between atom Y and atom C (2.48 Å) is larger than that between atom Nb and atom C (2.13 Å), reflecting the influence of the Janus asymmetry on both in-plane and out-of-plane structural properties.

### 3.2 Phononic and thermodynamic properties

Fig. 3 displays the calculated phonon spectra together with the corresponding total and projected phonon density of states (PhDOS) for the three monolayers: Fig. 3(a) for Nb_2_C, Fig. 3(b) for Y_2_C, and Fig. 3(c) for the j-YNbC. Each monolayer has a three-atom primitive unit cell, giving rise to nine phonon branches, all of which exhibit real frequencies. This confirms the dynamical stability of all three monolayers. The calculated phonon dispersions for Nb_2_C and Y_2_C are in close agreement with previously published theoretical results,^44,52^ further supporting the reliability of the present computational approach. The acoustic phonon modes consist of the longitudinal acoustic (LA), transverse acoustic (TA), and flexural acoustic (ZA) branches, with the ZA branch exhibiting the characteristic quadratic dispersion in the long-wavelength limit. The optical spectrum contains six vibrational modes: the in-plane TO and LO vibrations, the out-of-plane ZO vibration, and their corresponding antisymmetric counterparts (TO′, LO′, and ZO′). These classifications are illustrated in Fig. 3. As listed in Table 3, the average atomic masses (*M̄*) of the three monolayers are very close to each other. Therefore, one would expect their phonon frequency ranges to be similar. However, Nb_2_C exhibits a higher frequency range compared to the other two structures. This is attributed to its smaller *a*, which leads to stronger bonds and consequently higher phonon frequencies. A closer inspection of the projected PhDOS in Fig. 3 reveals distinct contributions from different atomic species. At low frequencies, corresponding to the acoustic phonon modes, the PhDOS is dominated by vibrations of the heavier transition-metal atoms. In contrast, at higher frequencies, within the range of the LO, TO, and ZO optical branches, the lighter carbon atoms exhibit the dominant contribution. These differences arise primarily from the fact that the transition metal atoms are much heavier than the carbon atoms.

Calculated vibrational mode frequencies, infrared (IR) and Raman-active modes [cm^−1^], average atomic mass in the unit cell (*M̄*) in atomic mass unit [amu], and average acoustic Debye temperature (*θ*_D_) [K] for Nb_2_C, Y_2_C, and j-YNbC monolayers

**Table:** 

Structure	E_g_	A_1g_	A_2u_	E_u_	*M̄*	*θ*_D_	Ref.
Y_2_C	129.2	205.9	224.7	387.8	63.27	196.72	This work
Nb_2_C	163.23	268.1	572.78	627.3	65.94	256.25	This work
	167.2	269	575.8	633.4	65.94	257.9	52

**Table:** 

Structure	E	A_1_	A_1_	E	*M̄*	*θ*_D_	Ref.
j-YNbC	148.88	217.40	520.66	531.77	64.60	221.55	This work

The optical phonon modes at the *Γ* point are characterized by the corresponding irreducible representations. For the Nb_2_C and Y_2_C, which belong to the *D*_3d_ point group, the optical modes are described by eqn (4a). In contrast, for the j-YNbC monolayer, the absence of inversion symmetry reduces the crystal symmetry to the *C*_3v_ point group, and the corresponding optical modes are given by eqn (4b), as illustrated in Fig. 3.4a
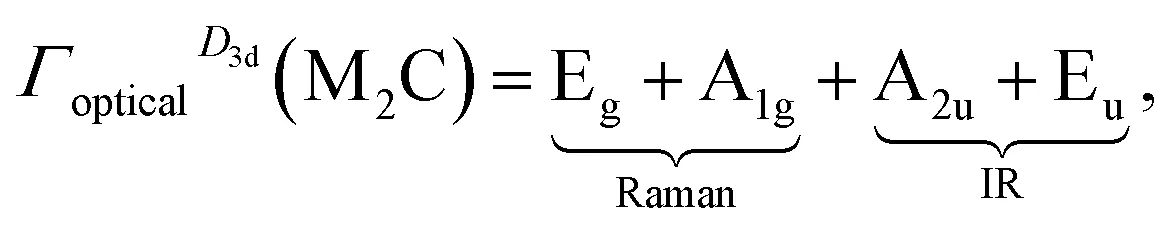
4b
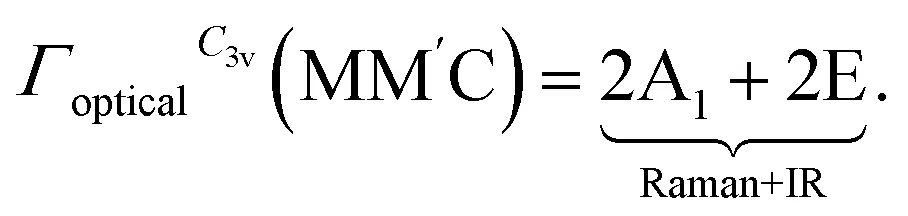


The corresponding atomic displacement patterns are shown schematically in Fig. 4, confirming the symmetry assignments and the direction of atomic displacements. For Nb_2_C and Y_2_C, E_g_ and A_1g_ are Raman-active, while A_2u_ and E_u_ are IR-active. Due to the broken inversion symmetry, all optical modes in j-YNbC are both Raman and IR active (I + R), which serves as a spectroscopic fingerprint of the Janus structure. In Nb_2_C and Y_2_C, the projected PhDOS (Fig. 3) reveals a clear separation: the lower-frequency optical branches arise predominantly from transition metal atoms, with negligible contribution from carbon. At higher frequencies, the optical phonon branches are primarily associated with vibrations of the carbon atoms. Accordingly, the Raman-active modes (E_g_ and A_1g_) originate from transition metal atoms, while the infrared-active modes (A_2u_ and E_u_) are dominated by carbon atoms. For the j-YNbC monolayer, a similar trend is observed: the lower-frequency optical modes originate from transition metal atoms, while carbon atoms make the dominant contribution to the higher-frequency optical modes.

**Fig. 4:**
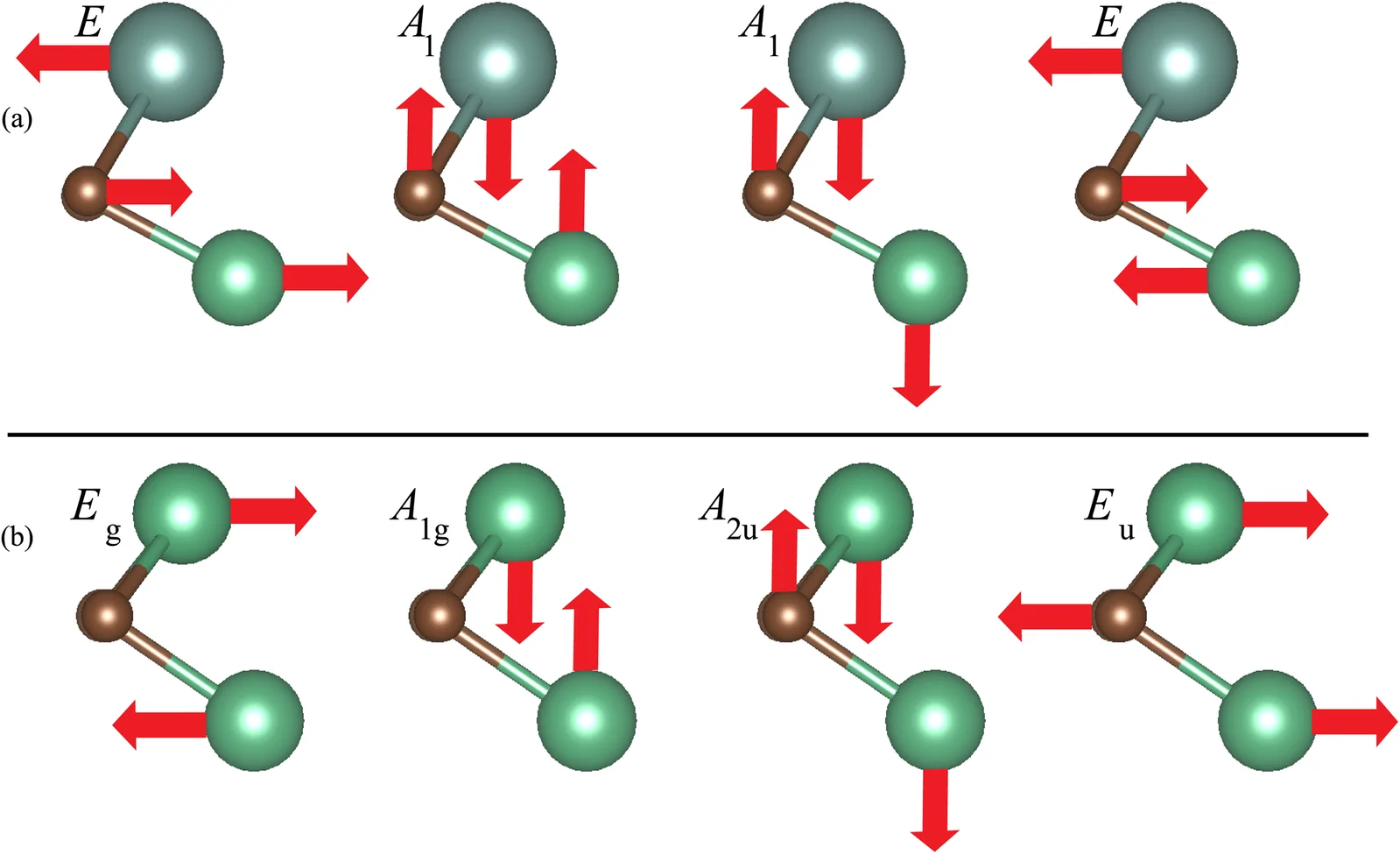
Schematic atomic displacement patterns of the *Γ*-point optical phonon modes for (a) the j-YNbC monolayer and (b) the parent Nb_2_C and Y_2_C MXenes. The corresponding mode symmetries are labeled according to the irreducible representations of the crystal point groups.

Having established the phonon properties in the previous section, we now proceed to evaluate the vibrational and finite-temperature thermodynamic properties of the monolayers. The first step involves calculating the average acoustic Debye temperatures (*θ*_D_) using the following relation:^52,53^5
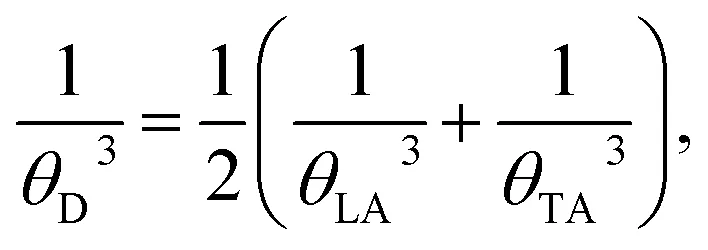
where, *θ*_*i*_ (*i* = LA, TA) is for the longitudinal and transverse acoustic branches and is defined as 
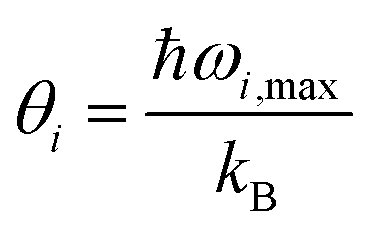
. Using these definitions, the above relationship can be rewritten as follows:^52^6
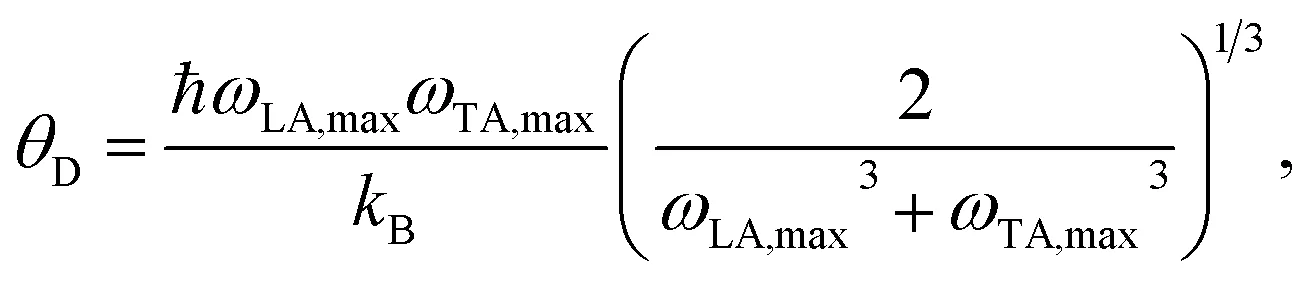
Here, ℏ(=*h*/2π) denotes the reduced Planck constant, *ω*_*i*,max_ represents the highest frequency of the *i*-th phonon branch at the Brillouin-zone boundary, and *k*_B_ denotes the Boltzmann constant. Table 3 summarizes the calculated *θ*_D_s. *θ*_D_ is a measure of lattice stiffness and bond strength. As listed in Table 3, *θ*_D_ of j-YNbC is 221.55 K, which lies between 196.72 K of Y_2_C and 256.25 K of Nb_2_C. Among the three monolayers, Nb_2_C exhibits the highest average acoustic Debye temperature. This can be attributed to its smaller *a*, which leads to stronger interatomic bonding and higher phonon frequencies compared to j-YNbC and Y_2_C.

The variation of the thermodynamic quantities with temperature was evaluated within the harmonic approximation. Fig. 5 presents four panels: (a) heat capacity at constant volume (*C*_V_), (b) vibrational entropy (*S*_th_), (c) internal energy (*E*_int_), and (d) Helmholtz free energy (*F*_vib_). Fig. 5(a) shows that *C*_V_ increases with temperature and gradually approaches the Dulong–Petit limit of about 9*R* = 74.8 J mol^−1^ K^−1^ at high temperatures. Among the three monolayers, Y_2_C exhibits the highest heat capacity, followed by j-YNbC and then Nb_2_C. The inset further shows that this ordering is already established in the low-temperature regime and remains consistent over the entire temperature range. The positive *C*_V_ values throughout the investigated temperature range are consistent with the expected finite-temperature thermodynamic behaviour of all considered layers. As shown in Fig. 5(b), the vibrational entropy *S*_th_ quantifies the number of thermally accessible phonon states. Its monotonic increase with temperature reflects the progressive activation of vibrational modes. The observed ordering 
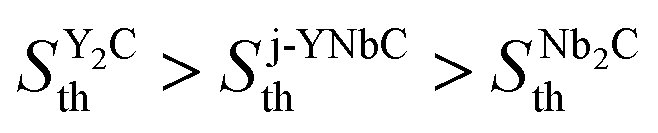
 is consistent with the corresponding trends in lattice stiffness and Debye temperature. The higher entropy of Y_2_C, which possesses the largest *a* and the softest lattice, indicates a greater density of accessible states at a given temperature, arising from its lower *θ*_D_. According to Fig. 5(c), *E*_int_ starts from a finite zero-point energy at *T* = 0 K, with Nb_2_C having the highest value (stiffest lattice), followed by j-YNbC and Y_2_C. At high temperatures, all curves become nearly linear, approaching the classical limit of 3*Nk*_B_*T*. This behaviour is consistent with the equipartition theorem, indicating that the vibrational modes become progressively populated and approach the classical regime. Fig. 5(d) shows that *F*_vib_ decreases monotonically with temperature. At *T* = 0 K, *F*_vib_ = *E*_int_ because the entropy *S* = 0. Hence, Nb_2_C has the highest *F*_vib_ and *E*_int_ at *T* = 0 K due to its larger zero-point energy, reflecting its higher lattice stiffness. The smooth decrease of *F*_vib_ without any abrupt change reflects the continuous temperature dependence of the vibrational free energy within the harmonic approximation.

**Fig. 5:**
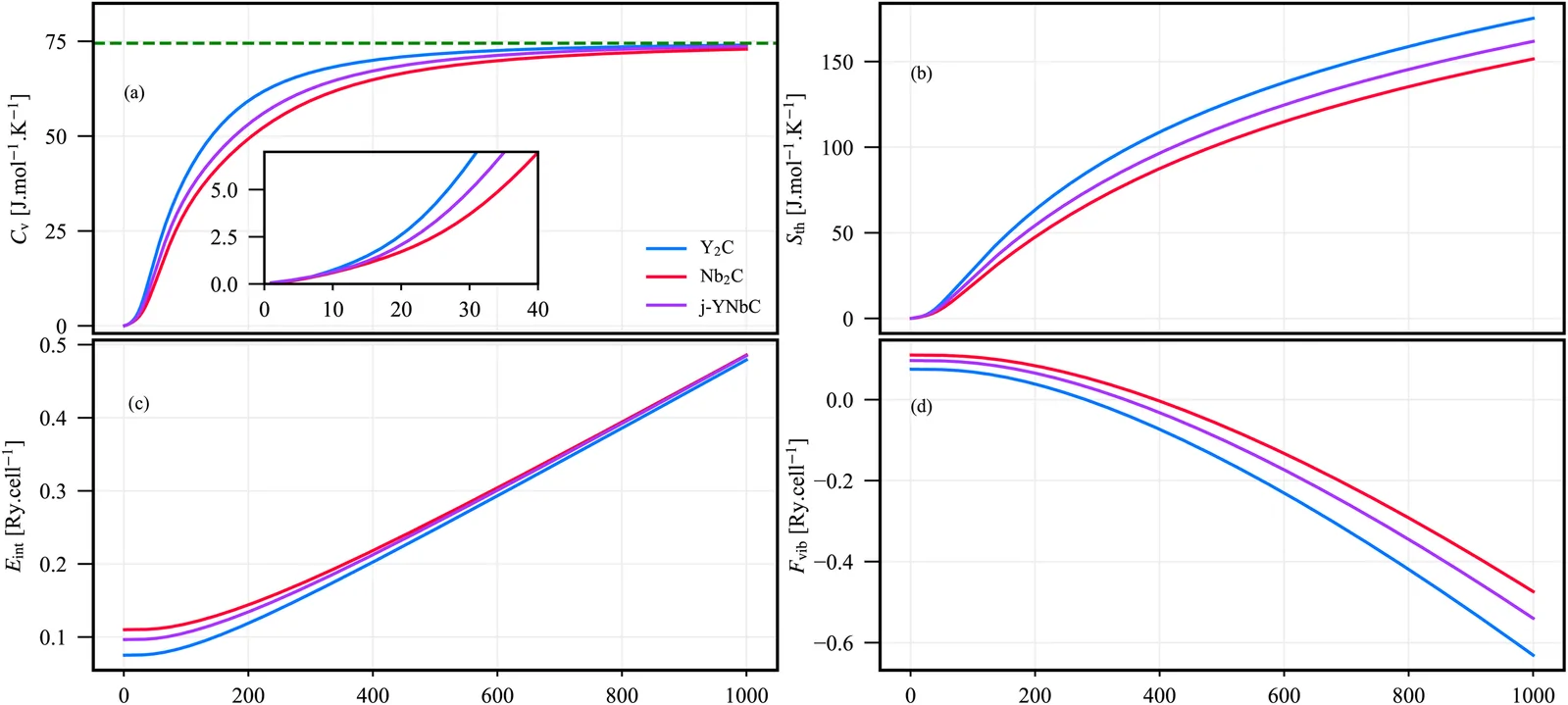
Temperature-dependent thermodynamic properties of the investigated monolayers calculated within the harmonic phonon approximation: (a) heat capacity at constant volume (*C*_V_), (b) vibrational entropy (*S*_th_), (c) internal energy (*E*_int_), and (d) Helmholtz free energy (*F*_vib_).

### 3.3 Mechanical properties

The calculated elastic constants were used to investigate the mechanical properties and mechanical stability of the monolayers. The generalized Hooke's law describes the stress–strain relation in the linear elastic regime, *σ̂* = *Ĉε̂*, with *Ĉ* denoting the elastic stiffness tensor, whereas *σ̂* and *ε̂* correspond to the stress and strain tensors, respectively. Following the standard Voigt notation (1: *xx*, 2: *yy*, 6: *xy*), the elastic constants *C*_11_, *C*_12_, *C*_22_, and *C*_66_ fully characterize the 2D hexagonal systems. Using this notation, the above tensor relation reduces to the following matrix form:7
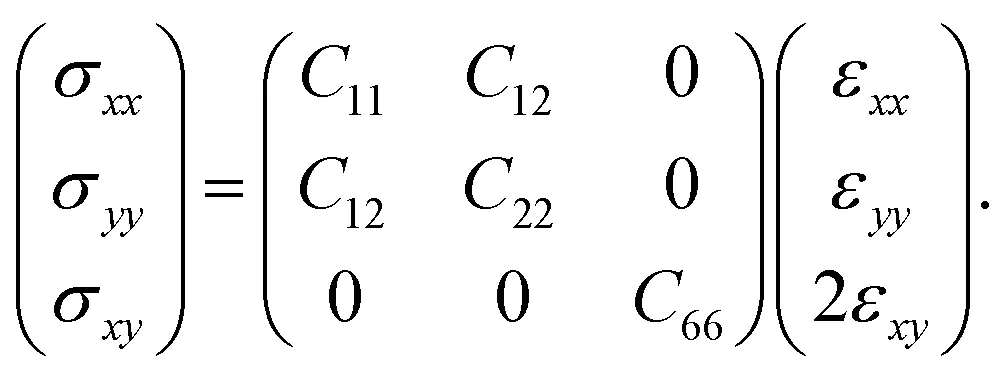


In this relation, *σ* and *ε* denote the stress and strain components, respectively. The strain was quantified using *ε* = (*a* − *a*_0_)/*a*_0_, where *a* is the lattice constant under strain and *a*_0_ is its equilibrium value. Within the small-strain regime, the strain energy is defined as *E*_s_ = *E* − *E*_0_, where *E* and *E*_0_ denote the total energies of the strained and equilibrium structures, respectively. In the small-strain limit, the strain energy can be written as:^52^8
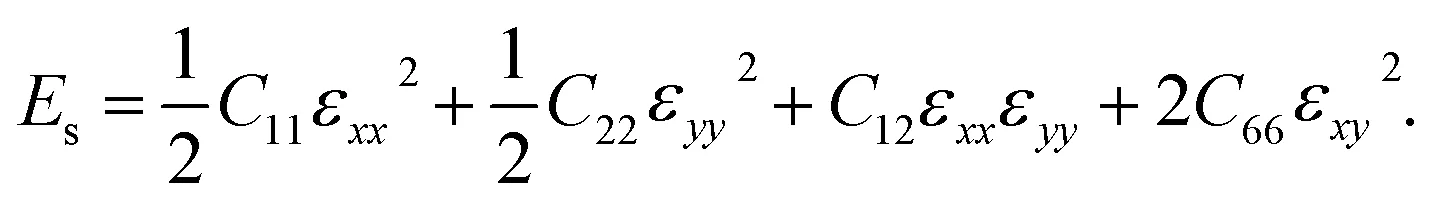


The elastic constants were derived from the strain energy using:9
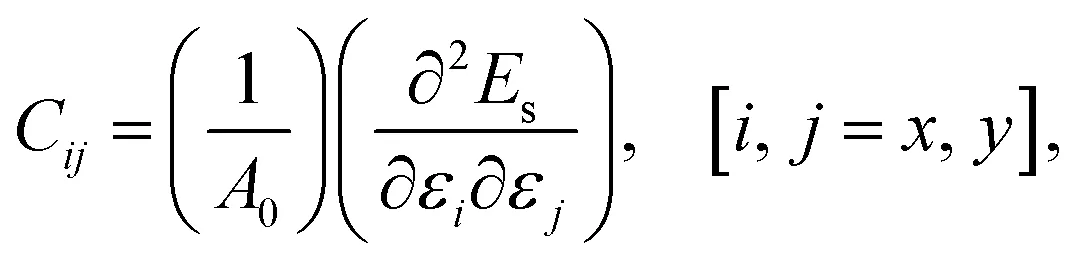
where *A*_0_ corresponds to the equilibrium area of the relaxed monolayer. Using the above relations, the elastic constants were derived by polynomial fitting of the energy-strain curves. To this end, the systems were subjected to uniaxial and biaxial deformations ranging from −2% to +2% in steps of 0.5%. After each deformation step, the internal atomic coordinates were fully relaxed while the imposed in-plane lattice deformation was kept fixed, and the corresponding total energy was recorded. Fig. 6 shows the energy variation relative to the equilibrium energy (fitted by a polynomial surface) as a function of strain for each compound. As evident from Fig. 6, the strain-energy surfaces of the investigated monolayers display different curvatures. These differences reflect their distinct mechanical stiffness and resistance to deformation and are consistent with the calculated elastic constants. To validate the accuracy of our computational method and to provide a basis for comparison, the calculated elastic constants for graphene, single-layer boron nitride (SL-BN), and the 2H phase of single-layer MoS_2_ (SL-MoS_2_) are summarized in Table 2, together with our results for Y_2_C, Nb_2_C, and j-YNbC. According to Table 2, our calculated values for the reference materials closely reproduce previously published values, supporting the reliability of the present calculations. For the hexagonal structures considered here, symmetry requires *C*_11_ = *C*_22_ and *C*_66_ = (*C*_11_ − *C*_12_)/2. For a 2D hexagonal system, the Born–Huang mechanical stability criteria require *C*_11_ > |*C*_12_| and *C*_66_ > 0. All investigated monolayers satisfy these criteria, indicating mechanical stability against small in-plane strains. The elastic constants allow the determination of several mechanical quantities. Specifically, the in-plane Young's modulus is *Y*_2D_ = (*C*_11_^2^ − *C*_12_^2^)/*C*_11_, the shear modulus is *G* = *C*_66_, the 2D bulk modulus is *B*_2D_ = (*C*_11_ + *C*_12_)/2, and *ν* denotes the Poisson's ratio, given by *ν* = *C*_12_/*C*_11_.^52,54–57^ All derived moduli are compiled in Table 2. The angular variation of the Poisson's ratio *ν*(*ϕ*) and Young's modulus *Y*_2D_(*ϕ*) can be expressed as:^58,59^10
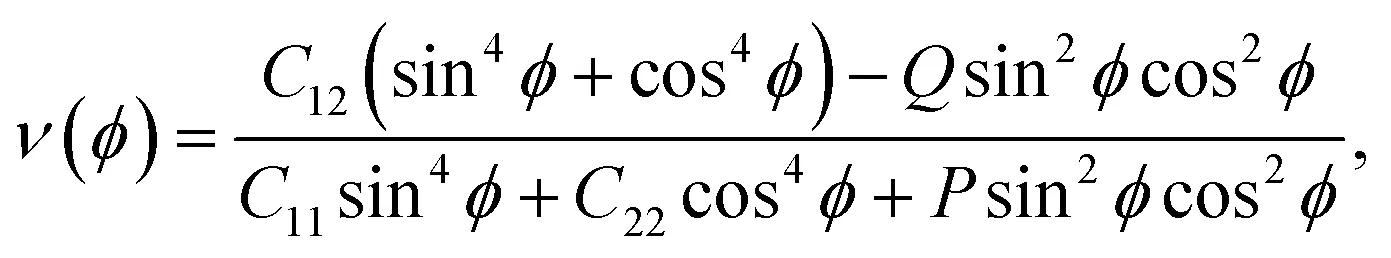
11
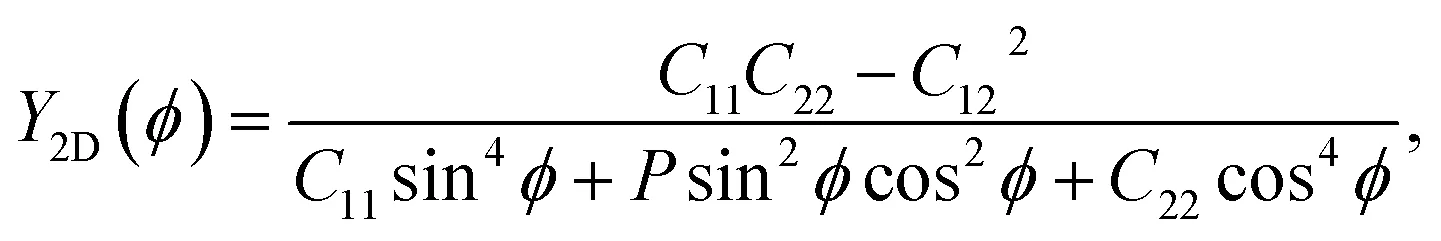
where 
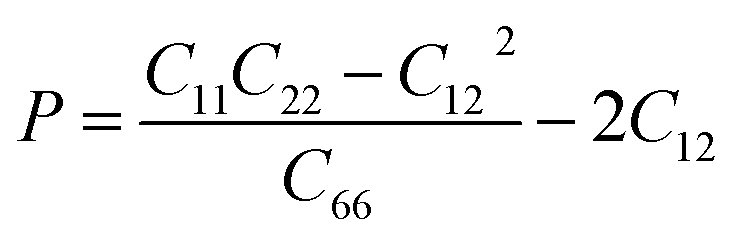
 and 
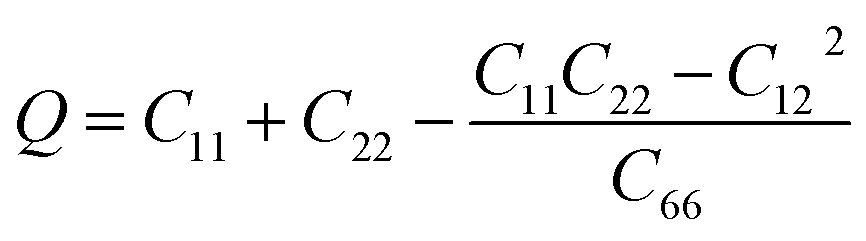
. From the calculated elastic constants, several additional physical parameters can be derived, including the longitudinal, transverse, and average acoustic velocities (*v*_l_, *v*_t_, and *v*_s_), together with the bending modulus (*D*) and the critical buckling strain (*ε*_c_). These quantities are obtained using the following relations:^55^12a
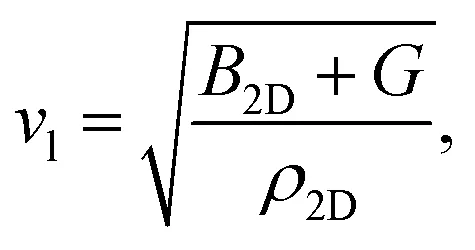
12b
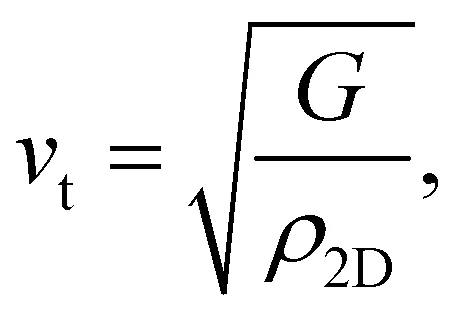
12c
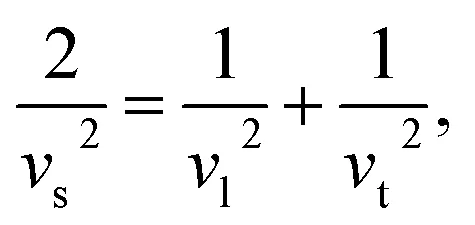
and:^57^13
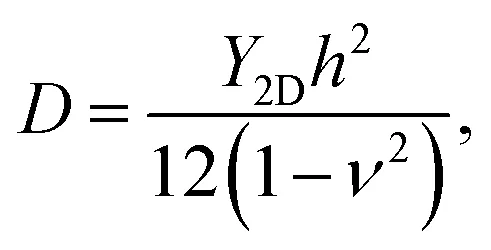
14
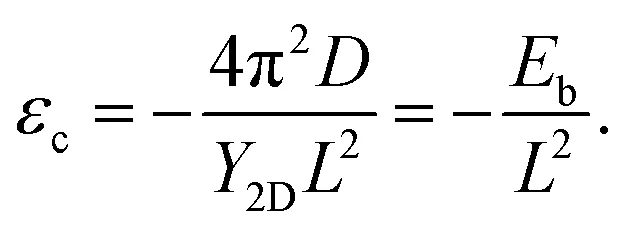


**Fig. 6:**
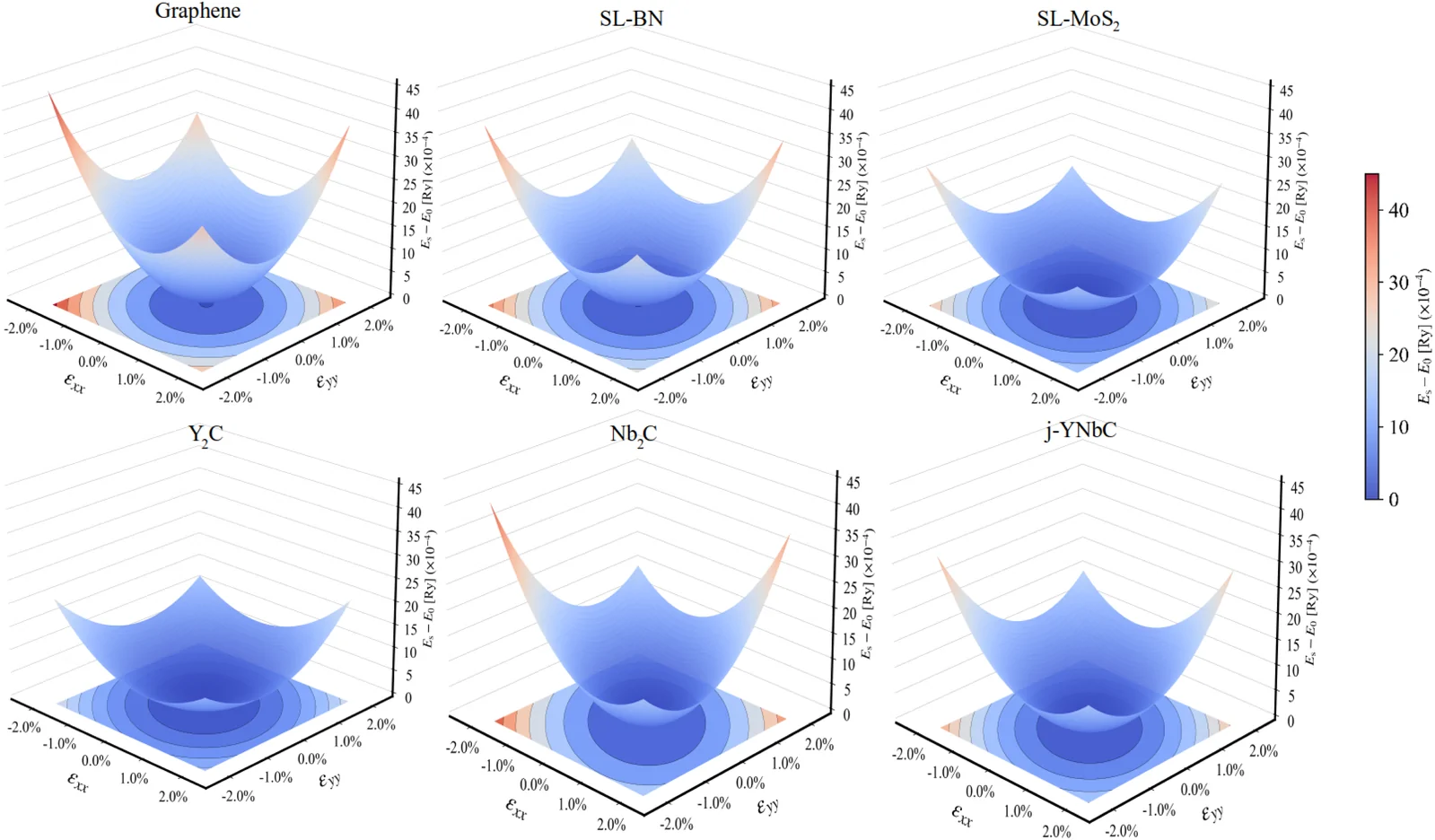
Strain-energy surfaces of the investigated systems under biaxial and uniaxial deformations. The elastic constants are obtained from polynomial fitting of the calculated energy-strain data.

In these relations, *ρ*_2D_ is the 2D mass density, defined as *ρ*_2D_ = *M*_cell_/*A*_0_, where *M*_cell_ refers to the total mass contained in the unit cell. For the hexagonal lattice, 
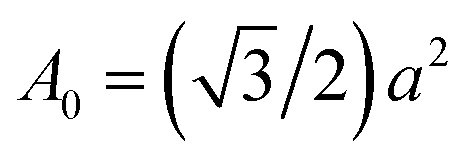
. The Euler buckling coefficient *E*_b_ is given by *E*_b_ = 4π^2^*D*/*Y*_2D_, where *h* and *L* denote the monolayer thickness and in-plane length, respectively, both expressed in angstroms (Å).

As listed in Table 2, the calculated *C*_11_ values show that j-YNbC 134.8 N m^−1^ is less stiff than Nb_2_C 172.54 N m^−1^ but significantly stiffer than Y_2_C 85.05 N m^−1^. The same trend is observed for the *Y*_2D_, *G* = *C*_66_, and *B*_2D_. This behaviour correlates with the lattice constants, which follow the order *a*_Nb_2_C_ < *a*_j-YNbC_ < *a*_Y_2_C_. The observed relationship suggests that a smaller lattice constant is associated with enhanced mechanical stiffness in the investigated systems. Although the j-YNbC exhibits lower mechanical performance than Nb_2_C in many aspects, its calculated elastic constants and derived mechanical moduli are well within the range of the well-known SL-MoS_2_. The Christensen criterion predicts a ductile-to-brittle transition in isotropic materials at a critical Poisson's ratio of approximately *ν* ≈ 2/7 ≈ 0.286.^54^ Based on this criterion, Nb_2_C with *ν* = 0.4 is classified as ductile, while Y_2_C with *ν* = 0.21 is brittle. The j-YNbC, with *ν* = 0.295, lies very close to the ductile–brittle transition according to this criterion. A clear correlation is observed between the Poisson's ratio and the bulk-to-shear modulus ratio (*B*/*G*): Nb_2_C has both the highest *ν* and the highest *B*/*G*, while Y_2_C has the lowest values for both quantities. This consistency indicates that the *B*/*G* ratio is in agreement with the classification obtained from the Christensen criterion.

The polar plots of *ν*(*ϕ*) and *Y*_2D_(*ϕ*) are presented in Fig. 7, with Fig. 7(a) and (b) corresponding to *ν*(*ϕ*) and *Y*_2D_(*ϕ*), respectively. The perfectly circular shape of these plots confirms that all three monolayers exhibit isotropic in-plane elastic properties. This behaviour is expected for materials with hexagonal symmetry, which generally exhibit isotropic in-plane mechanical responses. Furthermore, the radii follow the order Nb_2_C > j-YNbC > Y_2_C, consistent with the previously discussed trends in *C*_11_ and lattice constants.

**Fig. 7:**
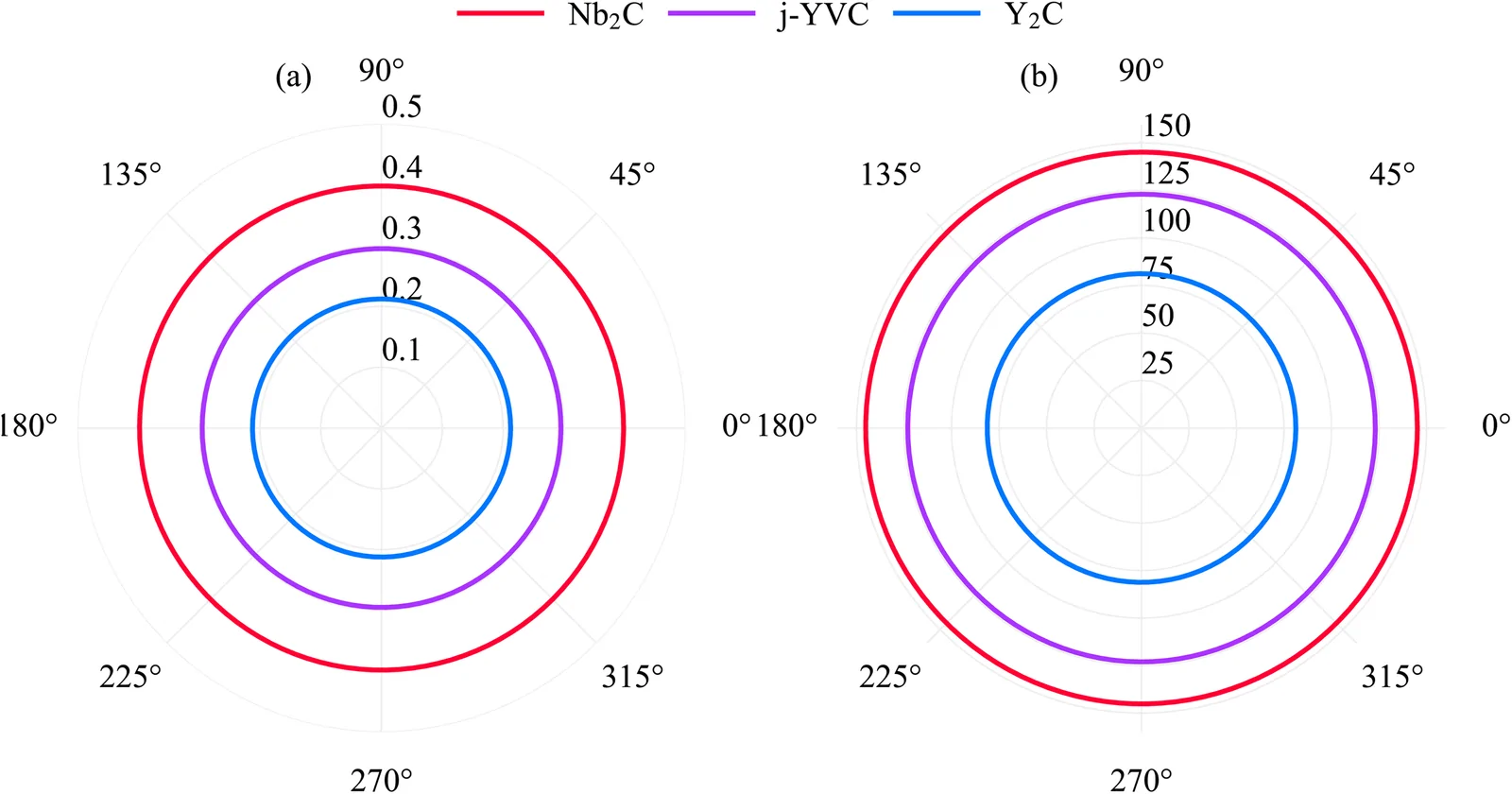
Angular dependence of (a) Poisson's ratio and (b) Young's modulus for the investigated monolayers.

*D* and *ε*_c_ characterize the out-of-plane mechanical behaviour. The calculated values are summarized in Table 4. As listed, *D* follows the order *D*_Y_2_C_ < *D*_j-YNbC_ < *D*_Nb_2_C_, consistent with their Young's moduli and thicknesses. *E*_b_ follows the opposite trend. This indicates that Y_2_C, despite having the lowest bending modulus, exhibits the highest resistance to buckling, while Nb_2_C, with the highest bending modulus, buckles more easily due to its significantly larger Young's modulus and smaller thickness. j-YNbC displays intermediate behaviour. These findings indicate that the buckling resistance of the investigated monolayers is governed by the combined effects of bending rigidity, in-plane stiffness, and thickness, with the latter playing a particularly important role in the present systems. Our calculated values lie between those of graphene (*D* ≈ 1.18 eV, *E*_b_ ≈ 2.17 Å^2^) and SL-MoS_2_ (*D* ≈ 6.74 eV, *E*_b_ ≈ 34.2 Å^2^). Consequently, all three monolayers exhibit higher buckling resistance than graphene but lower than SL-MoS_2_.

**Table 4:** Calculated longitudinal (*v*_l_), transverse (*v*_t_), and average (*v*_s_) sound velocities [m s^−1^], Euler buckling coefficient (*E*_b_) [Å^2^], bending modulus (*D*) [eV], and two-dimensional mass density (*ρ*_2D_) [×10^−6^ kg m^−2^] for graphene, SL-BN, SL-MoS_2_, Y_2_C, Nb_2_C, and the j-YNbC monolayer^a^

Structure	*v*_l_	*v*_t_	*v*_s_	*E*_b_	*D*	*ρ*_2D_	Ref.
Graphene	21 656	13 874	16 521	2.17	1.18	0.757	This work
	21 400	13 600	—	—	—	—	66
	—	—	—	2.2	1.2	—	57
SL-BN	19 610	12 227	14 673	2.215	0.967	0.756	This work
SL-MoS_2_	6616	4063	4897	34.2	6.74	3.03	This work
	6612	4141	—	—	—	3.3	55
	6600	4300	—	—	—	3.1*	67
	—	—	—	34.94	6.8	—	68
	—	—	—	—	6.62–13.24**	—	69
Y_2_C	5424	3404	4077	25.1	3.23	2.89	This work
Nb_2_C	6654	3650	4526	22.02	5.05	3.89	This work
j-YNbC	6271	3723	4528	23.7	4.62	3.42	This work

a *,** experimental data.

As listed in Table 4, Nb_2_C exhibits the highest *v*_l_, whereas j-YNbC shows the highest *v*_t_. The *v*_s_ values of Nb_2_C and j-YNbC are nearly identical and exceed that of Y_2_C. The calculated *v*_s_ values are significantly lower than those of graphene and are comparable to the corresponding values for SL-MoS_2_. These trends are consistent with the calculated elastic constants and *ρ*_2D_, from which the sound velocities were derived.

### 3.4 Electronic properties

To obtain a more comprehensive picture of the electronic structure beyond the conventional high-symmetry path, the energy dispersion of the bands near the Fermi level was mapped throughout the first Brillouin zone using a three-dimensional representation in the (*k*_*x*_ − *k*_*y*_ − *E*) space. As shown in Fig. 8, the conduction and valence bands are represented by red and blue surfaces, respectively, with the Fermi level set to zero. As evident from the Fig. 8, the conduction and valence bands cross the Fermi level in different regions of the Brillouin zone. The conduction-band minimum (CBM) and valence-band maximum (VBM) are marked in the three-dimensional representation by a red triangle and a blue circle, respectively. The corresponding two-dimensional projection of the first Brillouin zone is also presented at the bottom of the Fig. 8. In this projection, the red and blue points indicate the in-plane positions of the CBM and VBM, respectively, while the conventional high-symmetry path is included for comparison. The three-dimensional mapping across the entire Brillouin zone demonstrates that the CBM is located away from the conventional high-symmetry path, whereas the VBM is located at or very close to the *K* point. This finding indicates that a band structure calculated solely along the conventional high-symmetry path may not adequately capture the actual locations of the band extrema. Based on the band extrema identified from the full Brillouin-zone mapping, the band structures were calculated along a modified *k*-point path passing through the corresponding extrema. The band structures of Nb_2_C, Y_2_C, and j-YNbC are presented in Fig. 9(a), (b) and (c), respectively. These band structures were calculated to compare the PBE and PBE + SOC results, thereby enabling a more precise examination of the band-extrema locations and an assessment of the effect of spin–orbit coupling (SOC) on the electronic structure. Upon inclusion of SOC, a limited splitting of one band is observed around the *Γ* point in Nb_2_C, as shown in Fig. 9(a), at energies below the Fermi level. Since this splitting occurs below the Fermi level and away from the band edges, its direct influence on the low-energy electronic states is limited. This behaviour is also consistent with previously reported results for Nb_2_C.^52^ For Y_2_C, the inclusion of SOC induces only very minor changes in the band dispersion, as shown in Fig. 9(b). In j-YNbC, the modified path reveals a small indirect band overlap of approximately 14.6 meV between the valence- and conduction-band edges, indicating a semimetallic electronic character at the PBE level, as shown in Fig. 9(c). The PBE and PBE + SOC band structures appear nearly identical on the scale of the plotted energy range, and no pronounced SOC-induced splitting is observed near the Fermi level. Nevertheless, subtle differences between the PBE and PBE + SOC bands can be identified along certain portions of the band path. These subtle differences are consistent with the inequivalent chemical environments introduced by the intrinsic structural asymmetry of the Janus configuration. Despite the small visual differences between the PBE and PBE + SOC band structures, a quantitative analysis of the band extrema shows that the indirect band overlap increases from approximately 14.6 meV at the PBE level to 31.2 meV upon inclusion of SOC. Therefore, although SOC modifies the magnitude of the band overlap, it does not alter the qualitative electronic character of the system within the PBE framework. To further examine the sensitivity of the electronic structure to the choice of exchange-correlation functional, the band structures and total density of states (DOS) obtained using PBE and HSE are compared in Fig. 10. The comparison of the band structures in Fig. 10(a) shows that replacing PBE with HSE modifies the positions of the band edges identified above, namely the conduction-band minimum (CBM) and valence-band maximum (VBM), and eliminates the small band overlap predicted at the PBE level. In the HSE calculation, a finite indirect band gap of approximately 0.1 eV opens between the CBM and VBM, indicating that the system exhibits narrow-gap semiconducting behaviour at the HSE level. This gap is also clearly reflected in the DOS shown in Fig. 10(b), where a distinct zero-DOS region emerges between the valence- and conduction-band states. For a more detailed examination of the electronic states near the Fermi level, an enlarged view of the DOS is provided in the inset of Fig. 10(b), clearly resolving the DOS gap in the vicinity of the Fermi level.

**Fig. 8:**
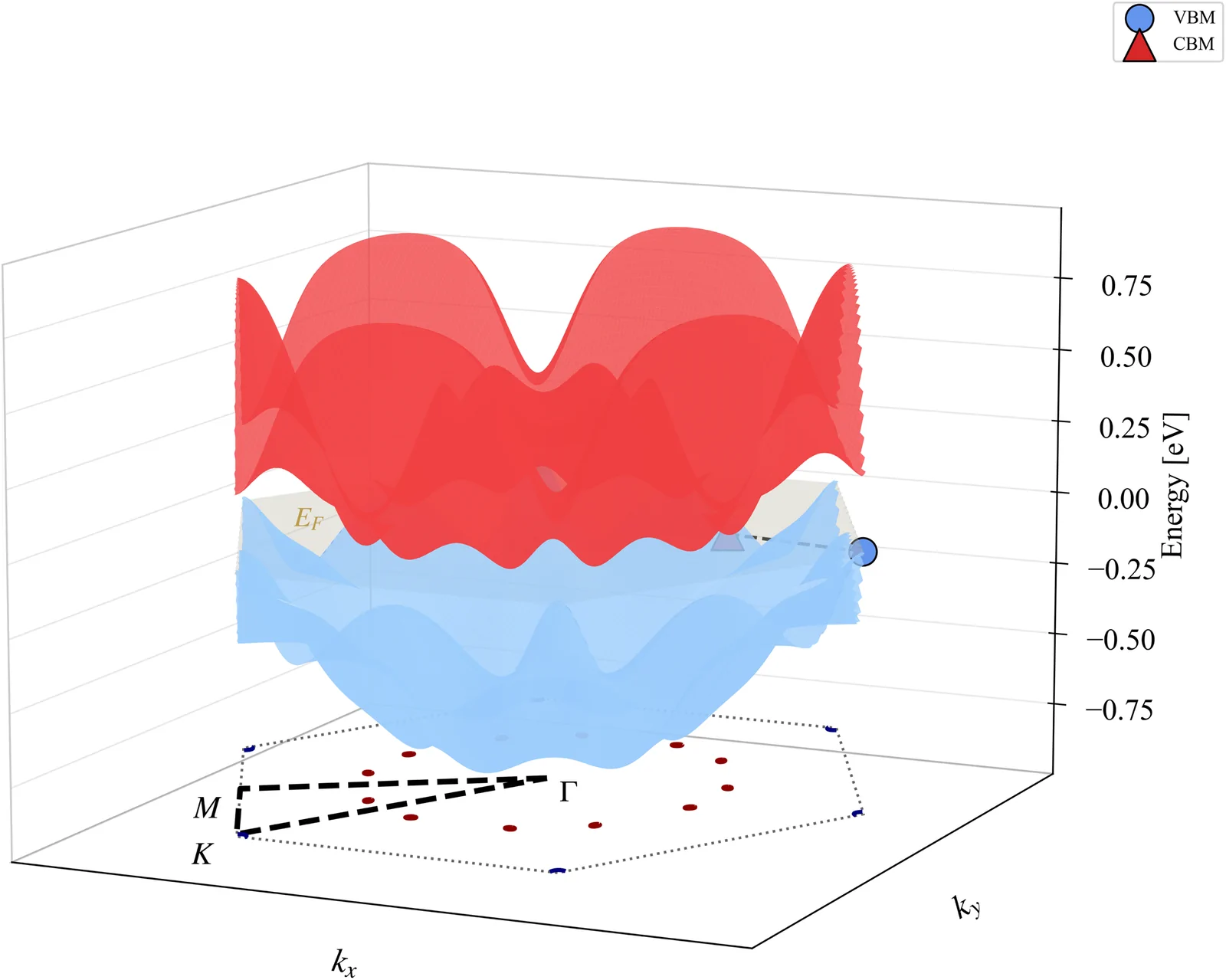
Three-dimensional mapping of the conduction and valence bands near the Fermi level for the j-YNbC Janus monolayer across the first Brillouin zone. The red triangle and blue circle denote the CBM and VBM, respectively, while the bottom projection shows their in-plane locations and the conventional high-symmetry path. The Fermi level is set to 0 eV.

**Fig. 9:**
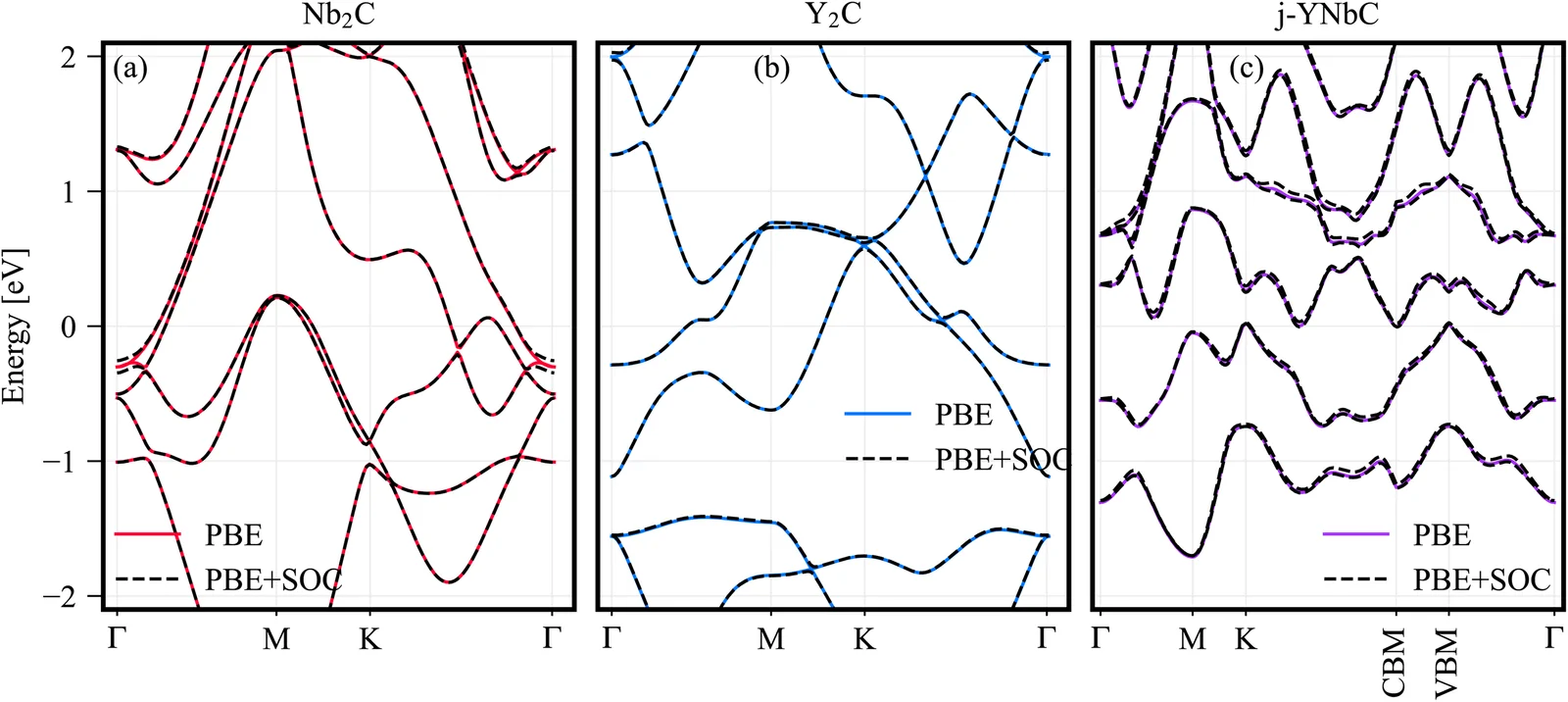
Electronic band structures of (a) Nb_2_C, (b) Y_2_C, and (c) j-YNbC. The PBE and PBE + SOC results are shown for comparison, with the Fermi level set to 0 eV.

**Fig. 10:**
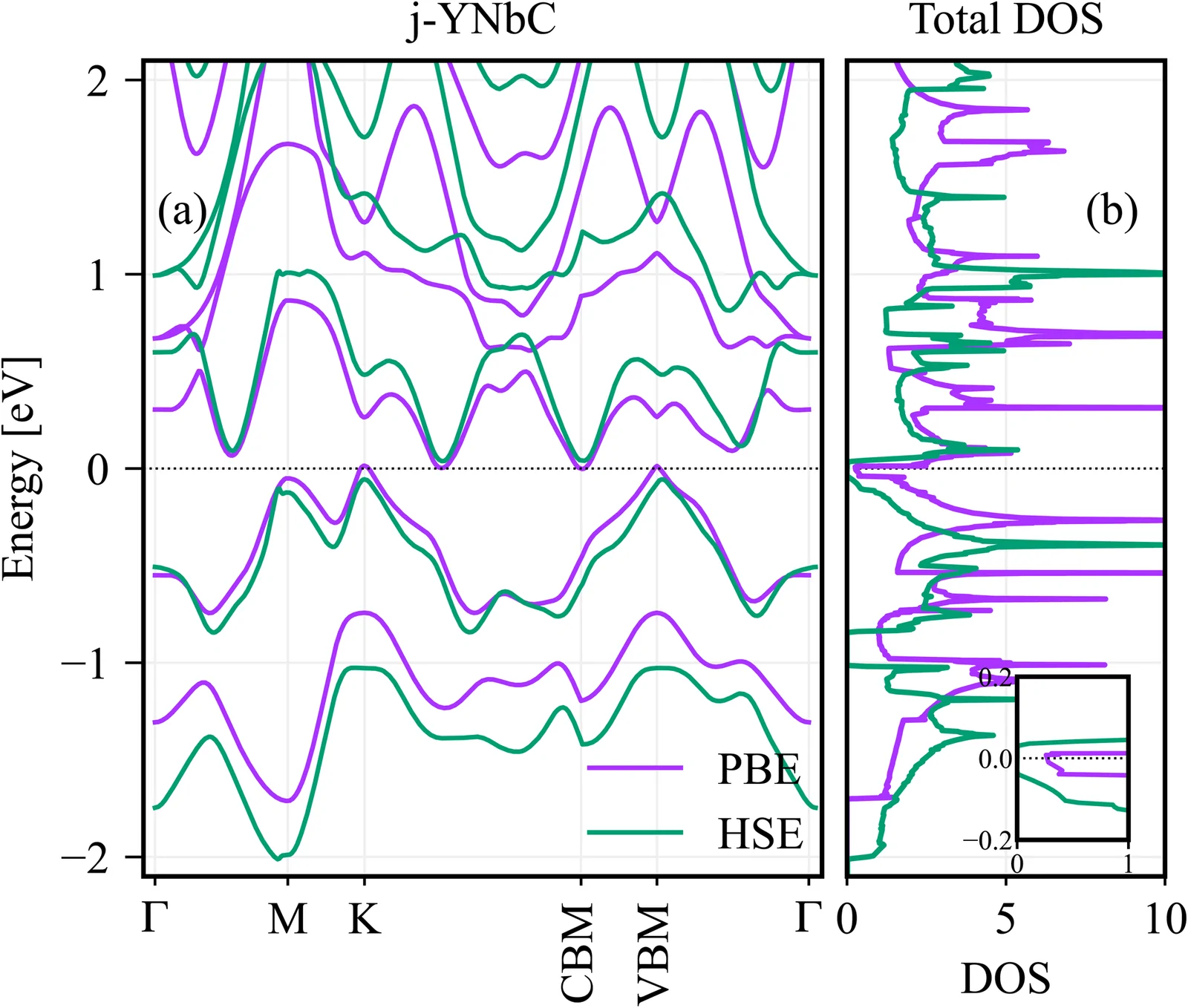
Electronic structure of the j-YNbC system obtained using PBE and HSE: (a) electronic band structures and (b) total density of states (DOS). The Fermi level is set to 0 eV.

The total density of states (DOS) and projected density of states (PDOS) of Nb_2_C, Y_2_C, and the j-YNbC monolayer are presented in Fig. 11(a_1_–a_3_), (b_1_–b_3_) and (c_1_–c_4_), respectively. For Nb_2_C and Y_2_C, the finite DOS at the Fermi level is consistent with their metallic electronic character. In contrast, the j-YNbC system exhibits a very small indirect band overlap of approximately 14.6 meV at the PBE level, as discussed above. To better resolve the electronic states in this narrow energy range, an enlarged view around the Fermi level is provided in the inset of Fig. 11(c_1_). The PDOS of Nb_2_C shown in Fig. 11(a_1_–a_3_) indicates that the electronic states around the Fermi level are dominated by the Nb-d orbitals, with a relatively smaller contribution from the C-p states. Similarly, the electronic states of Y_2_C shown in Fig. 11(b_1_–b_3_) are mainly contributed by the Y-d orbitals, while the contribution from the C-p states is comparatively smaller. These results indicate that the electronic states near the Fermi level in the parent MXenes are primarily governed by the transition-metal d-states.

**Fig. 11:**
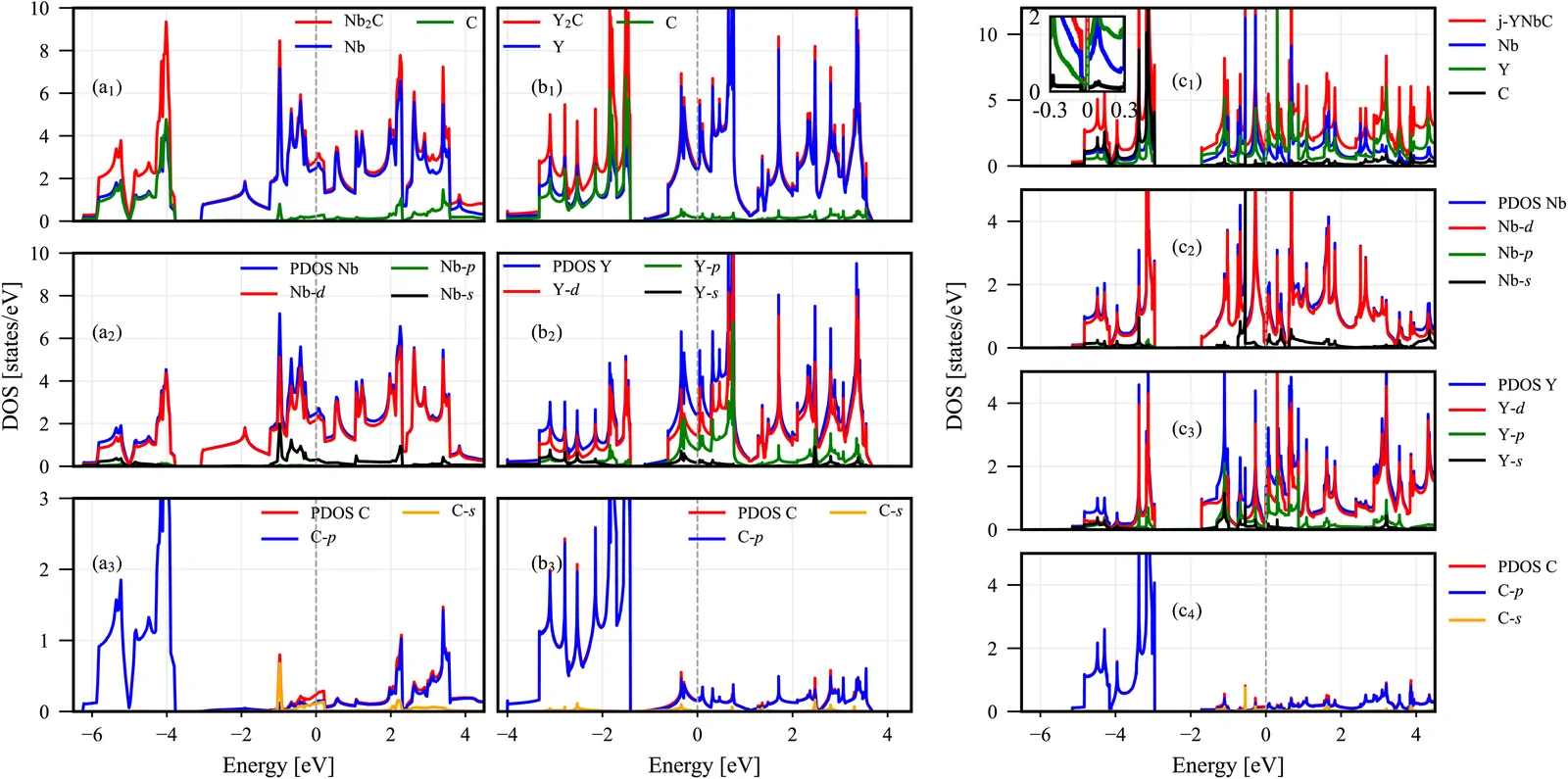
Total and projected density of states (DOS and PDOS) of (a_1_–a_3_) Nb_2_C, (b_1_–b_3_) Y_2_C, and (c_1_–c_4_) j-YNbC monolayers. The Fermi level is set to 0 eV and indicated by the vertical dashed line.

For the j-YNbC system, the PDOS shown in Fig. 11(c_1_–c_4_) reveals that the electronic states near the valence-band edge mainly originate from the transition-metal atoms, with Nb providing a larger contribution than Y. To quantitatively characterize the orbital contributions near the Fermi level, the orbital-resolved PDOS was integrated within the energy windows from −0.3 to 0 eV and from 0 to +0.3 eV relative to *E*_F_, representing the valence-band-like and conduction-band-like regions, respectively. In the valence-band-like region, Nb-4d states provide the dominant contribution (64.27%), followed by Y-4d (21.18%) and Y-p (5.87%) states. The contributions from C-p, C-s, Y-s, Nb-s, and Nb-p states are 2.99%, 1.76%, 1.75%, 1.63%, and 0.56%, respectively. In contrast, in the conduction-band-like region, Y-4d states become the dominant contribution (38.91%), followed by Nb-4d (28.01%) and Y-p (19.20%) states. The contributions from Nb-s, C-p, Y-s, C-s, and Nb-p states are 5.81%, 4.38%, 3.11%, 0.32%, and 0.25%, respectively. Thus, the quantitative PDOS analysis demonstrates a clear redistribution of the transition-metal-derived states across the Fermi level, with predominantly Nb-4d character below *E*_F_ and enhanced Y-derived contributions above *E*_F_. In contrast, the contributions from carbon states within the two energy windows remain relatively small.

The occupied states of j-YNbC extend from the Fermi level down to approximately −1.75 eV. This energy range is dominated by transition-metal states, which define the electronic structure in the vicinity of the band edges. A pronounced low-DOS region is then observed between approximately −1.75 and −3.85 eV, where the DOS is significantly reduced. At lower energies, from about −3.85 to −5.2 eV, the DOS increases again. Carbon p-states dominate this energy region, together with noticeable contributions from the d-orbitals of Nb and Y, with Nb providing the larger contribution. The overlap between these states indicates substantial M–C orbital hybridization. The larger contribution of Nb states in this energy range, together with the shorter Nb–C bond length, is consistent with a comparatively stronger Nb–C interaction. The asymmetric orbital contributions near the Fermi level are also reflected in the calculated band-edge alignment. At the PBE level, j-YNbC exhibits a very small indirect band overlap of approximately 14.6 meV, indicating a semimetallic character. In contrast, the HSE calculation removes this small overlap and yields a finite indirect band gap of approximately 0.1 eV, resulting in narrow-gap semiconducting behaviour. Thus, the redistribution of the nonequivalent Y- and Nb-derived states induced by the j-YNbC configuration is directly associated with the modified band-edge electronic structure, while the HSE treatment provides a more accurate description of the near-Fermi-level electronic states and opens a finite gap. Overall, the unequal orbital contributions of the two nonequivalent transition-metal atoms in j-YNbC reflect the chemical asymmetry introduced by the Janus configuration and contribute to the distinct electronic structure near the band edges.

To gain further insight into the charge distribution and surface electronic properties of the j-YNbC monolayer, Bader charge analysis and work-function calculations were performed. The Y, Nb, and C atoms exhibit net Bader charges of +0.906*e*, +1.048*e*, and −1.954*e*, respectively. These values indicate charge redistribution, with Nb exhibiting the largest positive Bader charge, followed by Y, while C exhibits a negative Bader charge corresponding to the largest electron accumulation. The near-zero total charge, Σ*q* ≈ 0, confirms the overall charge neutrality of the system. The unequal Bader charges of the nonequivalent Y and Nb sites are consistent with the chemically distinct local environments created by the Janus configuration. The planar-averaged electrostatic potential *V*(*z*) − *E*_F_ shown in Fig. 12 exhibits two well-defined vacuum plateaus, from which work functions of *Φ*_Nb_ = 4.241 eV and *Φ*_Y_ = 3.606 eV are obtained for the Nb- and Y-terminated surfaces, respectively. The resulting work-function difference, Δ*Φ* = 0.635 eV, provides quantitative evidence of the electrostatic inequivalence of the two surfaces. Such a difference in surface work functions indicates different energetic barriers for electron emission and can influence interfacial charge-transfer processes at the two surfaces. Overall, the unequal surface work functions, together with the asymmetric charge distribution revealed by the Bader analysis, provide complementary quantitative evidence of the intrinsic out-of-plane asymmetry induced by the Janus configuration.

**Fig. 12:**
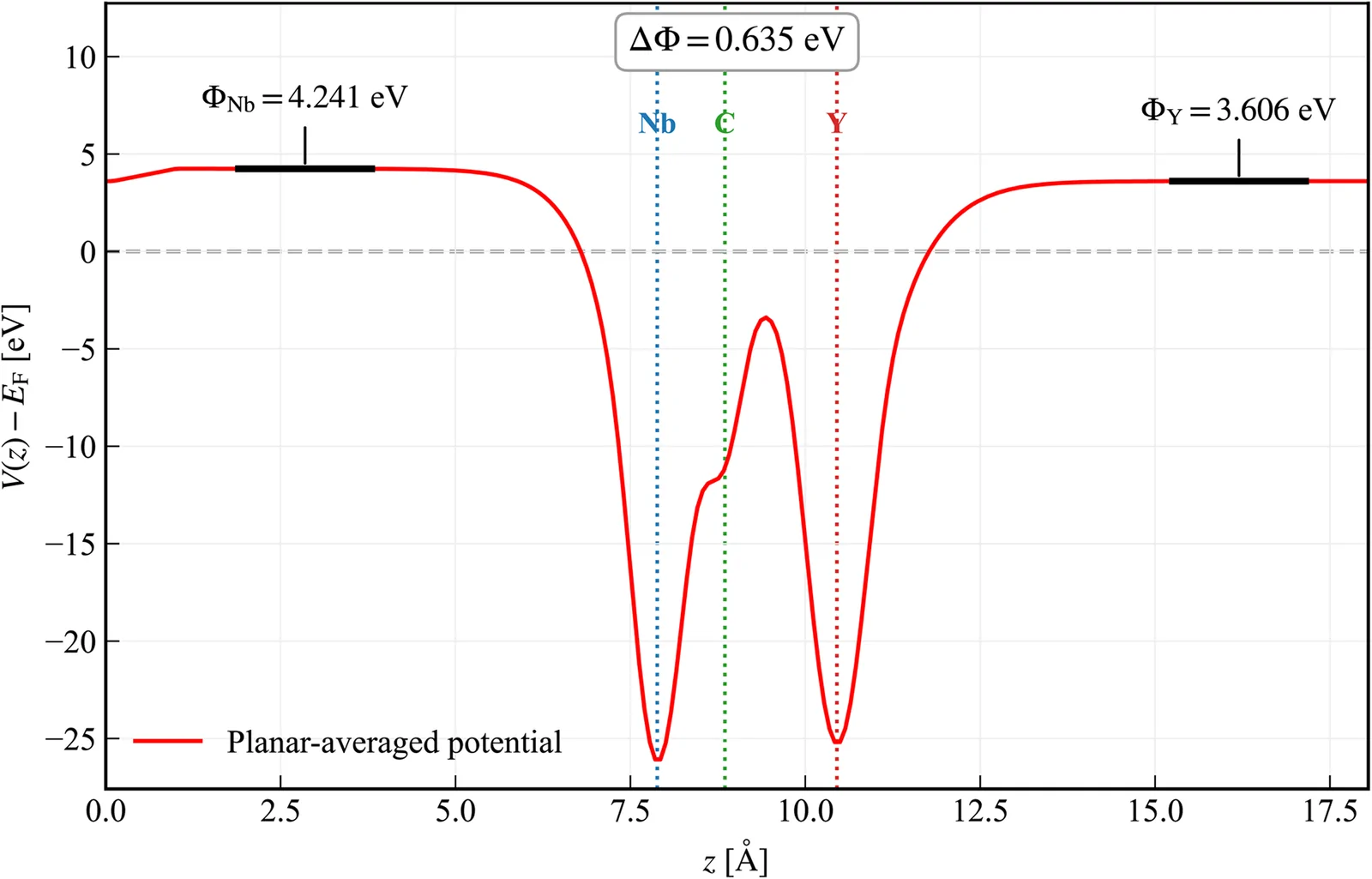
Planar-averaged electrostatic potential *V*(*z*) of the j-YNbC monolayer along the out-of-plane direction, showing the vacuum potential plateaus and the corresponding work functions of the two surfaces.

### 3.5 Thermoelectric properties

Fig. 13 depicts the variation of the thermoelectric transport parameters, namely the Seebeck coefficient (*S*), electrical conductivity (*σ*/*τ*_0_), electronic thermal conductivity (*κ*_e_/*τ*_0_), and power factor (PF), as a function of the chemical potential (*µ*) over the range −1 ≤ *µ* − *E*_F_ ≤ 1 eV at selected temperatures. For the two-dimensional systems considered here, the thermoelectric transport properties discussed below correspond to the in-plane components, for which the calculated *xx* and *yy* components are equivalent.

**Fig. 13:**
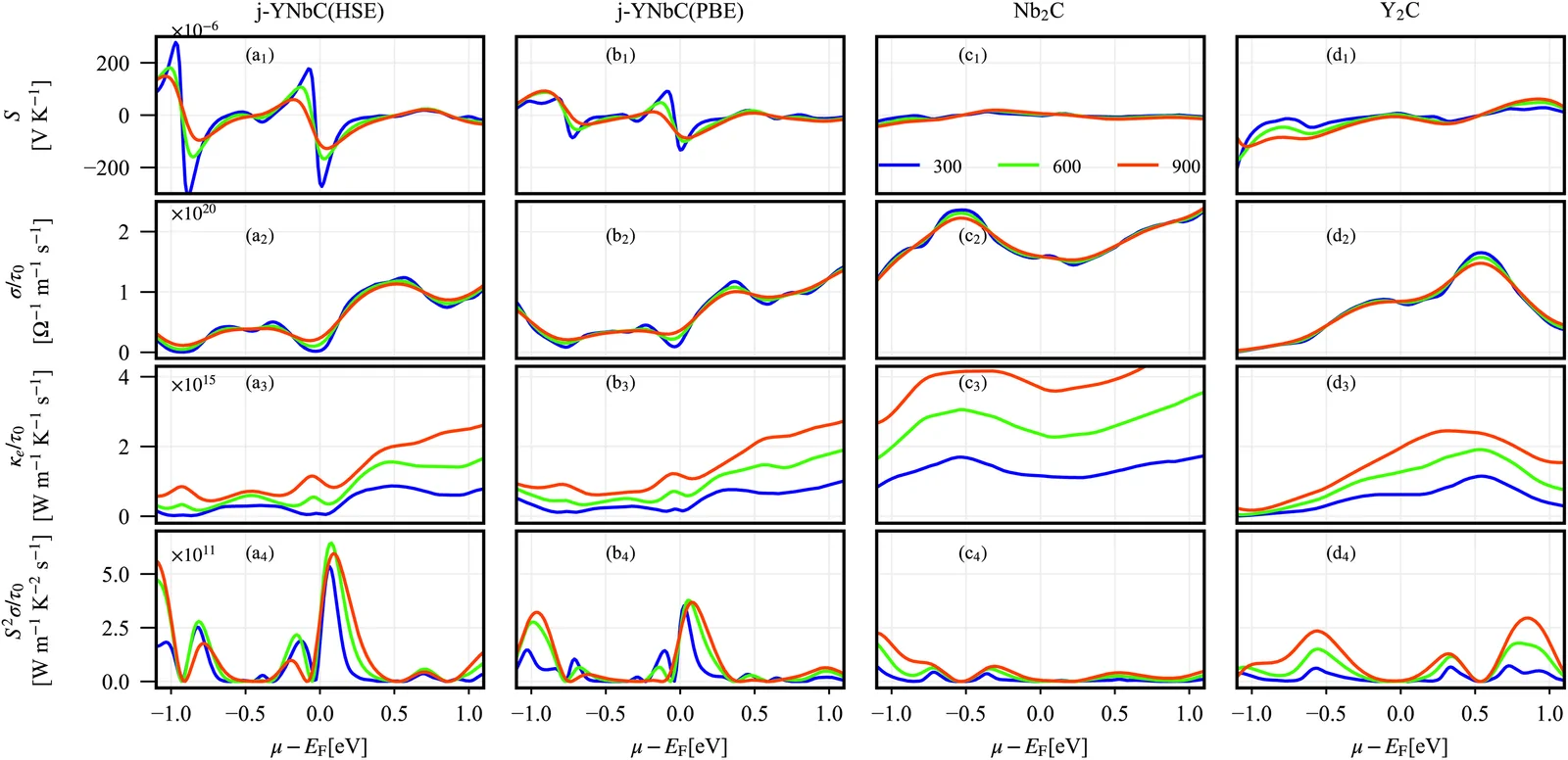
Calculated thermoelectric transport coefficients of j-YNbC, Nb_2_C, and Y_2_C as functions of chemical potential in the range −1 ≤ *µ* − *E*_F_ ≤ 1 eV at different temperatures. The first, second, third, and fourth rows correspond to the Seebeck coefficient (*S*), electrical conductivity normalized by the relaxation time (*σ*/*τ*_0_), electronic thermal conductivity normalized by the relaxation time (*κ*_e_/*τ*_0_), and power factor (PF = (*S*^2^*σ*)/*τ*_0_), respectively. The first and second columns present the HSE and PBE results for the j-YNbC monolayer, while the third and fourth columns correspond to Nb_2_C and Y_2_C MXenes, respectively.

As shown in Fig. 13(a_1_–d_1_), at 300 K, the HSE calculations yield Seebeck coefficients of approximately +177 µV K^−1^ and −273 µV K^−1^ on the negative and positive sides of the Fermi level, respectively, whereas the corresponding PBE values are approximately +91 µV K^−1^ and −133 µV K^−1^. These values substantially exceed those calculated for Nb_2_C and Y_2_C. For j-YNbC, the HSE calculations yield higher *S* than the PBE calculations, which is associated with the opening of a band gap and the accompanying modifications of the electronic structure near the band edges. A pronounced electron–hole asymmetry is observed, with the electron-doped side exhibiting a larger absolute value of *S*. This behaviour reflects differences in the electronic states around the valence- and conduction-band edges. With increasing temperature from 300 to 900 K, the magnitude of *S* gradually decreases, resulting in lower peak values. The decrease in *S* mainly results from the thermal broadening of the Fermi–Dirac distribution, which reduces the energy selectivity of carrier transport at elevated temperatures. Given the small HSE band gap of j-YNbC (*E*_g_ ≈ 0.1 eV), *k*_B_*T* is a substantial fraction of *E*_g_ at the temperatures considered (*k*_B_*T*/*E*_g_ ≈ 0.26, 0.52, and 0.78 at 300, 600, and 900 K, respectively), and bipolar (electron and hole) contributions to transport are therefore expected to become progressively important with increasing temperature. This is further supported by the Lorenz number, *L* = *κ*_e_/(*σT*), evaluated near the charge-neutral (intrinsic) chemical potential. Using the Sommerfeld value L_0_ = 2.44 × 10^−8^ W Ω K^−2^ as the Wiedemann–Franz reference, we obtain *L*/*L*_0_ ≈ 6.6, 3.7, and 2.6 at 300, 600, and 900 K, respectively, near mid-gap. The pronounced enhancement above *L*_0_ is consistent with an additional bipolar-diffusion contribution to *κ*_e_ in this narrow-gap system at elevated temperatures.^60,61^ The power-factor optima discussed below, however, occur near the band edges rather than mid-gap, where the contribution of one carrier type is expected to be dominant.

Fig. 13(a_2_–d_2_) present *σ*/*τ*_0_ as a function of *µ*. The electrical conductivity follows the order Nb_2_C > Y_2_C > j-YNbC, reflecting the high carrier availability in the metallic parent MXene monolayers and the reduced carrier availability in j-YNbC. For j-YNbC, the electronic character depends on the level of theory, with semimetallic behaviour obtained within PBE and semiconducting behaviour within HSE. Around *µ* − *E*_F_ = 0, the j-YNbC monolayer exhibits the lowest *σ*/*τ*_0_ among all investigated systems. Furthermore, the HSE calculations predict lower conductivity values than the PBE results in this region, owing to the opening of a band gap and the corresponding reduction in carrier concentration. As *µ* moves away from *E*_F_, *σ*/*τ*_0_ increases due to the enhanced availability of electronic states contributing to charge transport. For j-YNbC, the conductivity is slightly higher in the electron-doped region compared with the hole-doped region, indicating more efficient electron transport. The conductivity curves at different temperatures remain relatively close over most of the investigated chemical-potential range, while more noticeable deviations appear near *µ* − *E*_F_ = 0.

As shown in Fig. 13(a_3_–d_3_), *κ*_e_/*τ*_0_ closely follows the trend of the electrical conductivity, reflecting the common carrier-mediated origin of charge and heat transport. At comparable temperatures, Nb_2_C exhibits the highest *κ*_e_/*τ*_0_, followed by Y_2_C, while j-YNbC shows the lowest values. Around *µ* − *E*_F_ = 0, the HSE results for the j-YNbC exhibit the minimum electronic thermal conductivity among all investigated systems and remain lower than the corresponding PBE results. This reduction is associated with the low carrier concentration in this region. *κ*_e_/*τ*_0_ exhibits a similar trend, increasing as the chemical potential moves away from the low-carrier-concentration region, with larger values generally observed in the electron-doped region than in the hole-doped region, indicating a dominant contribution of electron carriers to electronic heat transport. Furthermore, *κ*_e_/*τ*_0_ increases with temperature for all investigated systems due to the enhanced contribution of thermally activated carriers.

The PF, shown in Fig. 13(a_4_–d_4_) and defined as PF = (*S*^2^*σ*)/*τ*_0_, provides an important measure of electronic thermoelectric transport properties by combining the effects of the *S* and *σ*/*τ*_0_. Despite its lower electrical conductivity compared with Nb_2_C and Y_2_C, the j-YNbC exhibits the highest PF values among all investigated systems. This enhancement is mainly driven by the pronounced *S*, which compensates for the reduced *σ*/*τ*_0_. Furthermore, the HSE calculations predict higher PF values than the PBE calculations, highlighting the importance of accurately describing the electronic structure for reliable predictions of thermoelectric transport properties. The most prominent power-factor peaks of the j-YNbC are consistently located in the n-type region and remain close to *µ* − *E*_F_ = 0 at all investigated temperatures. To further assess the doping requirements associated with the predicted thermoelectric performance, the carrier concentrations corresponding to the maximum in-plane power factor were evaluated at 300, 600, and 900 K. The resulting carrier concentrations are 7.22 × 10^13^, 1.46 × 10^14^, and 2.01 × 10^14^ cm^−2^, respectively. The maximum power factor is obtained at 600 K, corresponding to an optimal n-type carrier concentration of approximately 1.46 × 10^14^ cm^−2^. These values indicate that the predicted optimum thermoelectric performance corresponds to relatively high carrier modulation, within the 10^13^–10^14^ cm^−2^ range. Such carrier densities have been experimentally accessed in two-dimensional materials using electrostatic and electric-double-layer gating.^62^ In particular, n-type carrier densities up to 4 × 10^14^ cm^−2^ have been experimentally achieved in few-layer MoS_2_ using electrostatic doping.^63^

The present analysis focuses on the electronic contributions to thermoelectric transport in j-YNbC and its parent MXenes. A complete evaluation of the thermoelectric figure of merit (*ZT*), which additionally requires the lattice thermal conductivity (*κ*_L_), is beyond the scope of the present study.

## 4 Conclusion

The structural, vibrational, thermodynamic, mechanical, electronic, and thermoelectric properties of the j-YNbC monolayer were systematically investigated using first-principles calculations and benchmarked against those of the parent Nb_2_C and Y_2_C MXenes. The proposed Janus monolayer exhibits dynamical and mechanical stability, as evidenced by the absence of imaginary phonon modes and the satisfaction of the Born–Huang mechanical stability criteria, respectively. The negative cohesive energy indicates that the formation of the monolayer from isolated constituent atoms is energetically favorable, while the formation-energy analysis relative to the elemental reference states and the decomposition-energy analysis relative to the parent MXenes indicate that j-YNbC is a metastable phase. The corresponding finite-temperature thermodynamic response, evaluated within the harmonic phonon approximation, is physically consistent across the considered temperature range. The mechanical calculations reveal that j-YNbC possesses moderate in-plane stiffness and mechanical properties, with its overall mechanical properties lying between those of Nb_2_C and Y_2_C. The predicted elastic moduli, sound velocities, bending rigidity, and critical buckling strain further demonstrate the mechanical robustness of j-YNbC, indicating favorable mechanical characteristics for future applications. Electronic-structure calculations show that Nb_2_C and Y_2_C exhibit metallic behaviour, whereas j-YNbC exhibits semimetallic behaviour at the PBE level and narrow-gap semiconducting behaviour at the HSE level. The PBE calculation yields a small indirect band overlap of approximately 14.6 meV, whereas the HSE calculation removes this overlap and yields a finite indirect band gap of approximately 0.1 eV. The introduction of Janus asymmetry therefore substantially modifies the electronic structure of the metallic parent MXenes. The calculated thermoelectric transport coefficients demonstrate that j-YNbC exhibits higher Seebeck coefficients and power-factor values compared with Nb_2_C and Y_2_C. The favorable electronic thermoelectric transport response is primarily attributed to the finite band gap and the accompanying changes in the band-edge electronic states. As a consequence, j-YNbC exhibits a higher power factor despite its lower electrical conductivity relative to the metallic MXenes. These results demonstrate how structural asymmetry and bonding characteristics govern the electronic structure and, consequently, the thermoelectric response of the investigated monolayers.

Overall, the present results demonstrate that Janus engineering offers a promising strategy for designing MXene-based materials with improved electronic and thermoelectric properties. The combination of dynamical and mechanical stability, favorable mechanical properties, narrow-gap semiconducting behaviour, and favorable thermoelectric transport properties highlights the potential of j-YNbC for future MXene-based electronic and thermoelectric technologies.

## Conflicts of interest

There are no conflicts of declare.

## Data Availability

The dataset generated during this work is available at the following URL: https://doi.org/10.6084/m9.figshare.34542354.
